# Efficacy of hydroxychloroquine for post-exposure prophylaxis to prevent severe acute respiratory syndrome coronavirus 2 (SARS-CoV-2) infection among adults exposed to coronavirus disease (COVID-19): a structured summary of a study protocol for a randomised controlled trial

**DOI:** 10.1186/s13063-020-04446-4

**Published:** 2020-06-03

**Authors:** Ruanne V. Barnabas, Elizabeth Brown, Anna Bershteyn, R. Scott Miller, Mark Wener, Connie Celum, Anna Wald, Helen Chu, David Wesche, Jared M. Baeten, for the Hydroxychloroquine COVID-19 PEP Study Team

**Affiliations:** 1 Department of Global Health,International Clinical Research Center (ICRC), University of Washington, UW Box 359927, 325 Ninth Avenue, Seattle, WA 98104 USA; 2 Division of Allergy and Infectious Diseases, University of Washington, Seattle, WA USA; 3 Department of Epidemiolog, University of Washington, Seattle, WA USA; 4 Vaccine and Infectious Diseases Division, Fred Hutchinson Cancer Research Center, Seattle, WA USA; 5 Department of Biostatistics, University of Washington, Seattle, WA USA; 6 New York University School of Medicine, New York, NY USA; 7 Bill and Melinda Gates Foundation, Seattle, WA USA; 8 Department of Laboratory Medicine, University of Washington, Seattle, WA USA; 9 Certara, Princeton, NJ USA

**Keywords:** COVID-19, Randomised controlled trial, protocol, hydroxychloroquine, post-exposure prophylaxis, household contact, health care worker

## Abstract

**Objectives:**

Primary Objective

• To test the efficacy of Hydroxychloroquine (HCQ) (400 mg orally daily for 3 days then 200 mg orally daily for an additional 11 days, to complete 14 days) to prevent incident SARS-CoV-2 infection, compared to ascorbic acid among contacts of persons with SARS-CoV-2 infection

Secondary objectives

• To determine the safety and tolerability of HCQ as SARS-CoV-2 Post-exposure Prophylaxis (PEP) in adults

• To test the efficacy of HCQ (400 mg orally daily for 3 days then 200 mg orally daily for an additional 11 days, to complete 14 days) to prevent incident SARS-CoV-2 infection 2 weeks after completing therapy, compared to ascorbic acid among contacts of persons with SARS-CoV-2 infection

• To test the efficacy of HCQ to shorten the duration of SARS-CoV-2 shedding among those with SARS-CoV-2 infection in the HCQ PEP group

• To test the efficacy of HCQ to prevent incident COVID-19

**Trial design:**

This is a randomized, multi-center, placebo-equivalent (ascorbic acid) controlled, blinded study of HCQ PEP for the prevention of SARS-CoV-2 infection in adults exposed to the virus.

**Participants:**

This study will enroll up to 2000 asymptomatic adults 18 to 80 years of age (inclusive) at baseline who are close contacts of persons with polymerase chain reaction (PCR)-confirmed SARS-CoV-2 or clinically suspected COVID-19 and a pending SARS-CoV-2 PCR test. This multisite trial will be conducted at seven sites in Seattle (UW), Los Angeles (UCLA), New Orleans (Tulane), Baltimore (UMB), New York City (NYU), Syracuse (SUNY-Upstate), and Boston (BMC).

***Inclusion criteria***

Participants are eligible to be included in the study only if all of the following criteria apply:

1. Men or women 18 to 80 years of age inclusive, at the time of signing the informed consent
2. Willing and able to provide informed consent
3. Had a close contact of a person (index) with known PCR-confirmed SARS-CoV-2 infection or index who is currently being assessed for COVID-19

Close contact is defined as:

- Household contact (i.e., residing with the index case in the 14 days prior to index diagnosis or prolonged exposure within a residence/vehicle/enclosed space without maintaining social distance)
- Medical staff, first responders, or other care persons who cared for the index case without personal protection (mask and gloves)

4. Less than 4 days since last exposure (close contact with a person with SARS-CoV-2 infection) to the index case
5. Access to device and internet for Telehealth visits
6. Not planning to take HCQ in addition to the study medication

***Exclusion criteria***

Participants are excluded from the study if any of the following criteria apply:

1. Known hypersensitivity to HCQ or other 4-aminoquinoline compounds
2. Currently hospitalized
3. Symptomatic with subjective fever, cough, or shortness of breath
4. Current medications exclude concomitant use of HCQ
5. Concomitant use of other anti-malarial treatment or chemoprophylaxis, including chloroquine, mefloquine, artemether, or lumefantrine.
6. History of retinopathy of any etiology
7. Psoriasis
8. Porphyria
9. Known bone marrow disorders with significant neutropenia (polymorphonuclear leukocytes <1500) or thrombocytopenia (<100 K)
10. Concomitant use of digoxin, cyclosporin, cimetidine, amiodarone, or tamoxifen
11. Known moderate or severe liver disease
12. Known long QT syndrome
13. Severe renal impairment
14. Use of any investigational or non-registered drug or vaccine within 30 days preceding the first dose of the study drugs or planned use during the study period

**Intervention and comparator:**

Households will be randomized 1:1 (at the level of household), with close contact participants receiving one of the following therapies:

•HCQ 400 mg orally daily for 3 days then 200 mg orally daily for an additional 11 days

•Placebo-like control (ascorbic acid) 500 mg orally daily for 3 days then 250 mg orally daily for 11 days

**Main outcomes:**

The primary outcome of the study is the incidence of SARS-CoV-2 infection through day 14 among participants who are SARS-CoV-2 negative at baseline by randomization group.

**Randomisation:**

Participants will be randomized in a 1:1 ratio to HCQ or ascorbic acid at the level of the household (all eligible participants in 1 household will receive the same intervention). The randomization code and resulting allocation list will be generated and maintained by the Study Statistician. The list will be blocked and stratified by site and contact type (household versus healthcare worker).

**Blinding (masking):**

This is a blinded study.

HCQ and ascorbic acid will appear similar, and taste will be partially masked as HCQ can be bitter and ascorbic acid will be sour.

The participants will be blinded to their randomization group once assigned. Study team members, apart from the Study Pharmacist and the unblinded statistical staff, will be blinded. Laboratory staff are blinded to the group allocation.

**Numbers to be randomised (sample size):**

The sample size for the study is *N*=2 000 participants randomized 1:1 to either HCZ (*n*=1 000) and ascorbic acid (*n*=1 000).

**Trial status:**

Protocol version: 1.2

05 April 2020

Recruitment is ongoing, started March 31 and anticipated end date is September 30, 2020.

**Trial registration:**

**ClinicalTrials.gov, Protocol Registry Number:** NCT04328961

Date of registration: April 1, 2020, retrospectively registered

**Full protocol:**

The full protocol is attached as an additional file, accessible from the Trials website (Additional file 1). In the interest in expediting dissemination of this material, the familiar formatting has been eliminated; this Letter serves as a summary of the key elements of the full protocol.

## Supplementary information

**Additional file 1.** Full study protocol.

## Data Availability

Data from the study will be available from the study oversight committee on request ( rbarnaba@uw.edu ). The final dataset will be available through open access.

