# Supplementary material for: Efficacy of hydroxychloroquine for post-exposure prophylaxis to prevent severe acute respiratory syndrome coronavirus 2 (SARS-CoV-2) infection among adults exposed to coronavirus disease (COVID-19): a structured summary of a study protocol for a randomised controlled trial
Source: Trials. 2020 Jun 3;21:475. doi: 10.1186/s13063-020-04446-4 (PMC7268961; doi:10.1186/s13063-020-04446-4)
Supplement: Supplementary file 1 — Additional file 1. Full study protocol. [file 13063_2020_4446_MOESM1_ESM.pdf]

About this Model Protocol

This model document is a protocol template created to align institutions engaged in evaluating hydroxychloroquine (HCQ) therapy for coronavirus disease (COVID-19) post-exposure prophylaxis (PEP). It contains the following:

- Sections written in black text are mandatory and must be consistent across the participating institutions to maintain scientific integrity of the project
- *Sections written in italics are optional and may be omitted as required (e.g., based on institutional needs and practices)*
- A site-specific protocol addendum template (Appendix 3) is provided to capture information that is specific to a given study site

These materials are provided “AS IS” WITHOUT WARRANTY OF ANY KIND, EITHER EXPRESSED OR IMPLIED, INCLUDING, BUT NOT LIMITED TO, THE IMPLIED WARRANTIES OF MERCHANTABILITY, FITNESS FOR A PARTICULAR PURPOSE, OR NON-INFRINGEMENT. Authors and sponsors of this document do not accept any responsibility for any loss of any kind including loss of revenue, business, anticipated savings or profits, loss of goodwill or data, or for any indirect consequential loss whatsoever to any person using these materials or acting or refraining from action as a result of the information contained in these materials. Any party using these materials bears sole and complete responsibility for ensuring that the materials, whether modified or not, are suitable for the particular use and are accurate, current, commercially reasonable under the circumstances, and comply with all applicable laws and regulations.

Nothing in this model document should be construed to represent or warrant that persons using this document have complied with all applicable laws and regulations. All individuals and organizations using this document bear responsibility for complying with the applicable laws and regulations for the relevant jurisdiction.

CLINICAL STUDY PROTOCOL

Protocol Title:

Efficacy of Hydroxychloroquine for Post-exposure Prophylaxis (PEP) to Prevent Severe Acute Respiratory Syndrome Coronavirus 2 (SARS-CoV-2) Infection Among Adults Exposed to Coronavirus Disease (COVID-19): A Blinded, Randomized Study

Compound:

Hydroxychloroquine

Short Title:

Hydroxychloroquine for COVID-19 PEP

Protocol Version and Approval Date:

Version 1.2; 09 April 2020

Protocol Registry Number:

NCT04328961

Regulatory Agency Identifying Number(s):

Not applicable

Sponsor Name and Legal Registered Address:

University of Washington
Roosevelt Way NE, Seattle, WA 98115

Funding Agency and Legal Registered Address:

Bill & Melinda Gates Foundation
1300 I St NW, Washington, DC 20005

Co-Principal Investigators:

Dr. Ruanne V. Barnabas, University of Washington
Phone: +1 206 520 3813; Email: rbarnaba@uw.edu

Dr. Anna Bershteyn, NYU School of Medicine
Phone: +1 408 309 9610; Email: anna.bershteyn@nyulangone.org

Sponsor Signatory Name and Title:

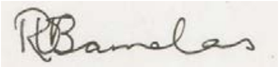

09 April 2020

Signature **Date**

Medical Monitor name and contact information is provided separately for each site.

Table of Contents

1. Synopsis 6

2. Schedule of Activities 10

3. Introduction 11
3.1. Background 11
3.2. Study Rationale 12
3.2.1. COVID-19 and Antiviral Approaches 12
3.2.2. Antiviral Effects of Chloroquine Analogues Against COVID-19 13
3.2.3. Rationale for Drug Selection 13
3.2.4. Rationale for Dosing Schedule 14
3.2.5. Rationale for Ascorbic Acid Control as a Comparator 15
3.2.6. Rationale for Enrolling the Index Case for the Index Case Sub-study 15
3.2.7. Rationale for Including Genetic Testing 15
3.3. Benefit/Risk Assessment 16

4. Objectives and Endpoints 17

5. Study Design 19
5.1. Overall Design 19
5.2. Participant and Study Completion 20

6. Study Population 21
6.1. Close Contact Participants 21
6.1.1. Inclusion Criteria 21
6.1.2. Exclusion Criteria 21
6.2. Index Cases 22
6.2.1. Inclusion Criteria 22
6.2.2. Exclusion Criterion 22
6.3. Screen Failures 22
6.4. Recruitment 22
6.5. Co-enrollment Guidelines 22

7. Treatments 23
7.1. Treatments Administered 23
7.2. Risks to the Participants 23
7.2.1. Risks Associated with PEP Administration with HCQ 23
7.2.2. Risks Associated with COVID-19 Surveillance and Quarantine 24
7.3. Strategies to Minimize Risk 24
7.3.1. Dose Selection 24
7.3.2. Management of Participants to Limit Risks of SARS-CoV-2 Transmission 24
7.4. Dose Modification and Toxicity Management 24
7.5. Method of Treatment Assignment 26
7.6. Blinding 26
7.7. Preparation/Handling/Storage/Accountability 26

7.8. Treatment Compliance 26
7.9. Concomitant Therapy 27
7.9.1. Prohibited Medications 27
7.9.2. Precautionary Medications....27
7.10. Treatment After the End of the Study....27

8. Discontinuation/Withdrawal Criteria 27
8.1. Discontinuation of Study Treatment....27
8.2. Withdrawal from the Study....28
8.3. Lost to Follow-up....28

9. Study Encounters 28
9.1. Close Contact Participants 29
9.1.1. Screening/Baseline Evaluation: Day 0/1 29
9.1.2. Day 2 Through Day 13 30
9.1.3. Day 14....30
9.1.4. Exit Contact (Day 28±3)....30
9.1.5. Participants who Develop COVID-19 During the Study....31
9.2. Index Cases 31
9.2.1. Baseline Evaluation: Day 1....31
9.2.2. Exit Contact (Day 28) 32
9.3. Participant Reimbursement....32

10. Study Assessments and Procedures....32
10.1. Efficacy Assessments 32
10.1.1. Mid-nasal Swab 32
10.1.2. Participant Survey....33
10.2. Adverse Events 33
10.2.1. Serious Adverse Events 33
10.2.2. AE and SAE Attribution to Study Medication 34
10.2.3. AE and SAE Reporting....34
10.2.4. Reporting of Use During Pregnancy....34
10.2.5. Reporting of Misuse of Drug 34
10.2.6. Treatment of Overdose 34
10.2.7. AEs Among Index Case Participants....34
10.3. Safety and AE Assessments....34
10.4. Dried Blood Spot Optional Sub-study....34
10.5. Genetic Testing....35
10.5.1. Samples Used for Genetic Testing....35
10.5.2. Genetic Testing Procedures 35
10.5.3. Genetic Information That Will Be Obtained 36
10.6. Biohazard Containment 36

11. Statistical Considerations....36
11.1. Sample Size Determination 36
11.2. Populations for Analyses 37
11.3. Statistical Analyses....37
11.3.1. Efficacy Analyses 38

11.3.2. Safety Analyses....38
11.3.3. Pharmacokinetic Analysis....38
11.3.4. Exploratory Exposure-Response Analyses....38
11.3.5. Exploratory Transmission Analyses....38
11.3.6. Combined Study Analysis....39

12. References....40

13. Appendices....43

Appendix 1: Abbreviations and Terms....43
Appendix 2: Study Governance Considerations....44
Appendix 3: Site-specific Protocol Addendum Template....48
Appendix 4: Hydroxychloroquine Label....51
Appendix 5: Physiologically Based Pharmacokinetic Modeling of Hydroxychloroquine Used for Post-exposure Prophylaxis....63
Appendix 6: Pharmacokinetic Sample Collection and Analysis....78

1. Synopsis

Protocol Title:

Efficacy of Hydroxychloroquine for Post-exposure Prophylaxis (PEP) to Prevent Severe Acute Respiratory Syndrome Coronavirus 2 (SARS-CoV-2) Infection Among Adults Exposed to Coronavirus Disease (COVID-19): A blinded, Randomized Study

Short Title:

Hydroxychloroquine for COVID-19 PEP

Rationale:

Post-exposure prophylaxis (PEP) is defined as taking antimicrobial medication after being exposed or potentially exposed to an infectious agent to prevent becoming infected.

PEP is routinely used for prevention of a variety of viral, bacterial, and parasitic infections, including influenza and human immunodeficiency virus. Based on experience with PEP for other infections, therapy should be started as soon as possible after a recent possible exposure.

Hydroxychloroquine (HCQ) is currently approved for the suppressive treatment and treatment of acute attacks of malaria due to several *Plasmodium* strains. It is also indicated for the treatment of discoid and systemic lupus erythematosus and rheumatoid arthritis. With the first Food and Drug Administration approval in 1955, safety and tolerability of HCQ are well described.

In vitro, HCQ displays antiviral activity against coronaviruses, including severe acute respiratory syndrome coronavirus 2 (SARS-CoV-2). Pharmacologic modeling based on observed drug concentrations and *in vitro* drug testing suggest that prophylaxis at approved doses could prevent SARS-CoV-2 infection and/or ameliorate viral shedding. Clinical trials of HCQ treatment for coronavirus disease (COVID-19) pneumonia are underway in China under separate protocol(s).

Objectives and Endpoints:

Objectives	Endpoints
Primary	
• To test the efficacy of HCQ (400 mg orally daily for 3 days then 200 mg orally daily for an additional 11 days, to complete 14 days) to prevent incident SARS-CoV-2 infection, compared to ascorbic acid among contacts of persons with SARS-CoV-2 infection	• Polymerase chain reaction (PCR)-confirmed SARS-CoV-2 infection (self-collected samples collected daily Day 1 through Day 14)

**Objectives**	**Endpoints**
Secondary	
• To determine the safety and tolerability of HCQ as SARS-CoV-2 PEP in adults	• Adverse events
• To test the efficacy of HCQ (400 mg orally daily for 3 days then 200 mg orally daily for an additional 11 days, to complete 14 days) to prevent incident SARS-CoV-2 infection 2 weeks after completing therapy, compared to ascorbic acid among contacts of persons with SARS-CoV-2 infection	• PCR-confirmed SARS-CoV-2 infection (self-collected samples collected daily Day 1 through Day 14 and collected 2 weeks after cessation of treatment [i.e., Day 28])
• To test the efficacy of HCQ to shorten the duration of SARS-CoV-2 shedding among those with SARS-CoV-2 infection in the HCQ PEP group	• SARS-CoV-2 viral shedding by PCR
• To test the efficacy of HCQ to prevent incident COVID-19	• PCR-confirmed COVID-19 diagnosis post start of HCQ therapy
Exploratory	
• *To conduct SARS-CoV-2 viral genotyping to assess the impact of HCQ on viral resistance to HCQ PEP*	• *Viral genotyping of SARS-CoV-2 in a subset of treated study participants who develop infection*
• To assess adherence to HCQ PEP	• Self-reported daily dosing *and study therapy concentration (if in sub-study)* in study participants
• *To evaluate the pharmacokinetics (PK) of HCQ by infection status*	• *HCQ blood concentration*
• *To assess if there is an association between PK of HCQ and the efficacy and safety endpoints evaluated*	• *HCQ blood concentration, tolerability, and efficacy*
• *To evaluate if SARS-CoV-2 viral load in the index case is associated with SARS-CoV-2 infection in the contact*	• *SARS-CoV-2 semiquantitative PCR and genotyping*

Objectives	Endpoints
• *To test the efficacy of HCQ to shorten the magnitude of SARS-CoV-2 shedding among those with SARS-CoV-2 infection in the HCQ PEP group*	• *Daily SARS-CoV-2 semiquantitative PCR*
• *To investigate the association of plausible genetic markers and susceptibility to SARS-CoV-2 infection and/or severity of COVID-19*	• *Rational gene candidates associated with SARS-CoV-2 infection and/or COVID-19 severity*

COVID-19: coronavirus disease; HCQ: hydroxychloroquine; PEP: post-exposure prophylaxis; SARS-CoV-2: severe acute respiratory syndrome coronavirus 2.

Overall Design:

This is a randomized, multi-center, placebo-equivalent (ascorbic acid) controlled, blinded study of HCQ PEP for the prevention of SARS-CoV-2 infection in adults exposed to the virus.

This study will enroll up to 2000 asymptomatic men and women 18 to 80 years of age (inclusive) at baseline who are close contacts of persons with polymerase chain reaction (PCR)-confirmed SARS-CoV-2 or clinically suspected COVID-19 and a pending SARS-CoV-2 PCR test. Eligible participants will be enrolled and randomized 1:1 to HCQ or ascorbic acid at the level of the household (all eligible participants in 1 household will receive the same intervention).

Participants will be counseled about the preliminary *in vitro* data on HCQ activity against SARS-CoV-2 and equipoise regarding efficacy in humans.

Participants may participate in a sub-study where they will be asked to provide a dried blood spot sample for therapy concentration and pharmacokinetics (PK) of HCQ as well as for SARS-CoV-2 antibody testing, if possible.

The index cases will be invited to participate in the index case sub-study.

An independent data and safety monitoring board (DSMB) will be convened for this study with expertise in COVID-19 or respiratory viruses, PEP, and emerging epidemics; DSMB will include a biostatistician. The purpose of the DSMB is to monitor the study for operational futility, social harms, and efficacy.

If additional data emerge on alternative effective agents for SARS-CoV-2 prophylaxis and/or data about HCQ as PEP, the protocol could be modified through an amendment to evaluate an alternative therapy as PEP. Alternatively, this platform study could be modified through an adaptive design to test the efficacy of new potential agents for COVID-19 PEP.

Number of Participants:

Up to 2000 eligible participants will be randomly assigned (at the level of household) 1:1 to study treatment (HCQ or ascorbic acid). *Up to 2000 index cases will be enrolled.*

Treatment Groups and Duration:

The duration of study participation will be approximately 28 days. Households will be randomized 1:1 (at the level of household), with close contact participants receiving one of the following therapies:

- HCQ 400 mg orally daily for 3 days then 200 mg orally daily for an additional 11 days
- Ascorbic acid 500 mg orally daily for 3 days then 250 mg orally daily for 11 days

After 14 days of treatment are completed, participants will be asked to participate in the study for an additional 14 (±3) days.

Eligible index cases will be asked to provide a mid-nasal swab at baseline to determine the viral load and complete a questionnaire at baseline and on Day 28 to report their clinical course.

2. Schedule of Activities

Procedure	Screening	Self-Quarantine							Exit Contact[b]
		Treatment Period							
	Day 0[a]	Day 1[a]	Day 2	Day 3	Days4 to 7	Day 8	Days9 to 13	Day 14	Day 28 (±3 days)
Informed consent	X	*X*[c]							
Demography	X	*X*[c]							
Past and current medical conditions, including known pregnancy and/or lactation status	X								
Concomitant medications	X								
Exposure to index case information	X								
Inclusion and exclusion criteria	X	*X*[c]							
Randomization	X								
Mid-nasal swab		X	X	X	X	X	X	X	X
Study therapy (HCQ or ascorbic acid)		##	##	##	#	#	#	#	
Daily Survey (including dosing and swab adherence, concomitant medications, and symptoms review)		X	X	X	X	X	X	X	
Exit Contact Survey (including concomitant medications, symptoms, etc.)									X
Adverse Event review		X	X	X	X	X	X	X	X
If in sub-study: dried blood spot sample for HCQ concentration and anti-SARS-CoV-2 antibodies (if possible)		*X*	*1 to 5 samples (no more than 1 per day) after dosing start*						*X*
Index case participant mid-nasal swab		*X*							
Index case participant questionnaire		*X*							*X*

EOS: end of study; HCQ: hydroxychloroquine; HIPAA: Health Insurance Portability and Accountability Act; SARS-CoV-2: severe acute respiratory syndrome coronavirus 2.

Note: ## denotes either 400 mg dose HCQ or 500 mg dose of ascorbic acid (depending on group assignment); # denotes either 200 mg dose HCQ or 250 mg dose of ascorbic acid (depending on group assignment).

[a] Screening and Day 1 evaluations will be conducted through a web-based screening tool, HIPAA-compliant video conference (Telehealth), telephone, or text messaging. Screening and Day 1 evaluations may occur on the same day.

[b] These evaluations will be conducted by telephone, text messaging, or through Telehealth.

[c] *Index case sub-study participants only.*

3. Introduction

This is a randomized, multi-center, placebo-equivalent (ascorbic acid) controlled, blinded study of the efficacy of hydroxychloroquine (HCQ) post-exposure prophylaxis (PEP) for the prevention of severe acute respiratory syndrome coronavirus 2 (SARS-CoV-2) infection in adults exposed to the virus.

Evaluations include safety and tolerability, SARS-CoV-2 viral shedding, and coronavirus disease (COVID-19) diagnosis. Two study groups (one active and one receiving ascorbic acid [vitamin C], to serve as a placebo-equivalent comparator) will be enrolled to assess a daily dosing regimen administered for 14 days, which appears to be the approximate incubation period of SARS-CoV-2 infection.

Up to 2000 eligible participants 18 to 80 years of age will be randomized (at the level of household) 1:1 to receive one of the following therapies:

- HCQ 400 mg orally daily for 3 days then 200 mg orally daily for an additional 11 days
- Ascorbic acid 500 orally daily for 3 days then 250 mg orally daily for 11 days

HCQ and ascorbic acid will appear similar, and taste will be partially masked as HCQ can be bitter and ascorbic acid will be sour.

During the study participants will perform the following:

- Collect mid-nasal swabs for viral detection for the primary trial endpoint
- Complete Surveys that will include questions about symptoms from both the drug regimen and virus infection, review of concomitant medications, and other pertinent topics
  Note: Participants who report respiratory or other febrile illness will be referred for assessment to their primary care provider.

During the first 14 study days, participants take medication, complete Surveys, and collect mid-nasal swab to assess symptoms and virus exposure. On Day 28, a final swab is collected and Survey completed. The duration of study participation will be approximately 28 days.

Up to 2000 eligible index cases for the COVID-19 PEP study will be invited to participate and asked to provide a baseline mid-nasal swab for SARS-CoV-2 testing and complete a questionnaire at baseline and on Day 28 to report the clinical course of their illness.

3.1. *Background*

SARS-CoV-2 is a coronavirus novel to the human population discovered in December 2019; it is currently the cause of a global pandemic [1,2,3]. The World Health Organization (WHO) named the novel coronavirus SARS-CoV-2 and the disease caused by SARS-CoV-2 COVID-19.

As of 08 March 2020, person-to-person transmission has occurred in China, across temperate Asia, Europe, and North America, and with sporadic cases in Africa and the southern hemisphere. Accurate reporting is limited by availability of diagnostic testing. The WHO declared the COVID-19 pandemic a Public Health Emergency of International Concern on 30 January 2020 [4], and the United States declared a national emergency on 13 March 2020 [5].

Most deaths and severe pneumonitis have occurred in the elderly or in persons with underlying pulmonary or cardiac comorbidities or diabetes. In healthy adults, including pregnant women, it can cause a febrile, self-limited pneumonia. Infection appears less symptomatic in children and younger adults [6]. Nevertheless, the burden of this pandemic to the global health and economic systems is expected to be substantial. No acquired immunity to this novel viral infection appears to exist in the human population globally, and no effective treatment or preventative agent is licensed at this time.

As with many infectious epidemics, household contacts, first responders, caregivers, and medical personnel attending persons with COVID-19 are at high risk of infection. The incubation time requires 14 days of quarantine for exposed individuals not wearing personal protective equipment [7], and on 03 March 2020, WHO declared a global shortage of personal protective equipment leaving doctors, nurses, and other frontline workers dangerously ill-equipped to care for COVID-19 patients [8]. Extensive absences from the care network and health system will degrade the ability to care not only for those with COVID-19 but also for routine healthcare issues as well.

PEP is defined as taking antimicrobial medication after potential exposure to an infectious agent to prevent becoming infected. PEP should be started as soon as possible after a recent possible exposure to the infectious agent [9]. At this time, there is no known effective prophylaxis for COVID-19 and thus there is equipoise for testing a non-active comparator (e.g., ascorbic acid) against a potentially active drug.

3.2. *Study Rationale*

3.2.1. *COVID-19 and Antiviral Approaches*

SARS-CoV-2 is a novel betacoronavirus of zoonotic origin, similar to the coronaviruses severe acute respiratory syndrome coronavirus (SARS-CoV) and Middle East respiratory syndrome coronavirus (MERS). Based on current evidence, case fatality rate for SARS-CoV-2 is about 3%, which is significantly lower than SARS-CoV (10%) and MERS-CoV (40%) [10]. However, SARS-CoV-2 has potentially higher transmissibility (R0: 1.4-5.5) than both SARS-CoV (R0: 2-5) and MERS-CoV (R0: <1) [10].

Our understanding of the viral pathogenesis of SARS-CoV-2 remains limited. However, it appears that the virus cell entry depends on binding of the viral spike (S) proteins to cellular receptors and on S protein priming by host cell proteases. SARS-CoV-2, like SARS-CoV, uses the same receptor angiotensin converting enzyme 2 (ACE2) on pulmonary epithelial cells for entry and the transmembrane serine protease 2 for S protein priming [11]. The receptor binding domain of lineage B betacoronaviruses is a single, continuous domain that contains all of the structural information necessary to interact with the host receptor. Fusion is mediated at the cell membrane, delivering the viral nucleocapsid inside the cell for subsequent replication. ACE2 expression is found in the lung epithelial cells, vascular endothelium, renal tubular epithelium, and epithelia of the small intestine. Viral shedding has been localized primarily to respiratory droplets and fecal samples [2].

Medications to treat and/or prevent SARS-CoV-2 need to inhibit aspects of the viral life cycle, ultimately blocking replication. Already-approved and available medications are ideal for

immediate evaluation for SARS-CoV-2 infection treatment and prevention. Two potential targets for anti-SARS-CoV-2 medications are viral polymerases and proteases [12]. Pilot clinical studies are already ongoing for SARS-CoV-2 using various repurposed antiviral medicines (https://www.who.int/emergencies/diseases/novel-coronavirus-2019/global-research-on-novel-coronavirus-2019-ncov). Similarities between SARS-CoV-2 with SARS-CoV and MERS suggest that antivirals with *in vitro* efficacy against SARS-CoV and MERS may be promising agents as SARS-CoV-2 PEP [12,13].

3.2.2. *Antiviral Effects of Chloroquine Analogues Against COVID-19*

Chloroquine (CQ) was discovered in 1934 by Bayer and was used in 1945 as an antimalarial to become one of the most prescribed drugs globally, prior to the emergence of widespread drug resistance in *Plasmodium falciparum* [14]. CQ was found to be effective against rheumatoid tenosynovitis in 1951 [15]. HCQ was licensed in the United States in 1955 as an antimalarial and as a drug for rheumatoid arthritis, and it was widely marketed for the latter due to a favorable safety profile with chronic use [16]. The mechanisms of action for HCQ for treatment of rheumatoid arthritis and other autoimmune diseases are still not fully understood despite widespread use over the past 60 years [17].

CQ and HCQ have been proposed as potential agents for treatment and prevention against other infectious agents beyond malaria [18,19]. The mechanism of action differs according to the pathogen: against intracellular bacteria and fungi by alkalinizing vacuoles containing the microorganisms, restoring the activity of other antibiotics, and against viral replication through alkalization of acidic organelles, namely endosomes, lysosomes, and Golgi vesicles.

CQ is effective *in vitro* against SARS-CoV coronavirus in Vero E6 cells with the EC_{50} ~8 μM [20] and had shown evidence of prevention activity in vivo [21]. Hence, these re-purposed drugs were obvious hits for testing against SARS-CoV-2. *In vitro* inhibition in Vero E6 cells against the novel coronavirus, SARS-CoV-2, has been published in recent weeks. Wang et al (2020) showed that the EC_{50} and EC_{90} for CQ in Vero E6 cells is 1.13 μM and 6.90 μM, respectively [22]. Yao et al (2020) showed that the EC_{50} for CQ treatment of infected cells at 48 hours was 5.47 μM, whereas HCQ appeared slightly more potent, with EC_{50} of 0.72 μM at 48 hours [23]. These levels appear to be within the range of exposures that could be achieved with standard HCQ treatment, and likely prophylaxis, due to concentrations of the drug achieved in the lung tissue [23]. No *in vitro* data in the lung epithelial cells are available nor are any animal model data.

Multiple observational and small Investigator-initiated COVID-19 pneumonia treatment trials using CQ, HCQ, and variety of other medications are ongoing in China (https://www.who.int/emergencies/diseases/novel-coronavirus-2019/global-research-on-novel-coronavirus-2019-ncov). Gao et al reported anecdotal efficacy of CQ as treatment for COVID-19–associated pneumonia [24].

3.2.3. *Rationale for Drug Selection*

The trial will test a daily regimen of HCQ for 14 days after exposure to SARS-CoV-2 (package insert Appendix 4). Daily dosing has the highest likelihood to achieve sustained required drug levels for viral inhibition, as shown in the physiologically based PK (PBPK) modeling

(Appendix 5). HCQ is commonly used daily in doses up to 600 mg of HCQ sulfate (465 mg base) per day for rheumatoid arthritis or systemic lupus erythematosus initially, with a usual maintenance dose 200 mg (155 mg base) for maintenance therapy. HCQ and CQ are both commonly used in a weekly dosing schedule for malaria chemoprophylaxis.

HCQ is associated with a better safety profile for daily and chronic use than CQ, including 5 decades of experience with use in these dose ranges in adults and the elderly. It is on the WHO Essential Medicines List for use in rheumatic disorders and is widely prescribed as an anti-inflammatory for rheumatoid arthritis, systemic lupus erythematosus, and other autoimmune syndromes. Based on the limited *in vitro* data available, HCQ appears to be slightly more potent than CQ against SARS-CoV-2 [23].

As the COVID-19 epidemic remains very fluid and new data are emerging from observational and clinical trials daily, this protocol is written to allow adaptation to incorporate additional medications beyond HCQ to be tested as PEP.

3.2.4. *Rationale for Dosing Schedule*

HQC is a long-acting drug with a terminal half-life of approximately 40 days. It is well absorbed, moderately protein bound, and will accumulate in tissues including the lung, heart, liver, and kidneys. It is typically given with a loading dose of approximately 2-fold the standard dose to accelerate achieving steady state drug concentrations [25]. Since the drug will be used in those with a recent high-risk exposure to SARS-CoV-2, it is desirable to achieve adequate drug levels quickly.

A PBPK model was built (SIMCYP simulator v.18) using physical and chemical parameters of HCQ obtained from the literature [26]; PK parameters (liver intrinsic clearance, fa, ka) were determined from clinical data [27]. This PBPK model was used to simulate HCQ concentrations in plasma and lung fluid following 5 proposed dosing regimens in order to select an optimal regimen for the Peking University Third Hospital's ongoing trial of HCQ in China. The combination of *in vitro* antiviral concentration-effect and predicted drug concentrations in this study were used to propose a loading dose of 400 mg HCQ twice a day on Day 1, followed by HCQ 200 mg twice a day on Day 2 through Day 5.

A second study (BYSY-DCTC-CPPO-HCQ-PBPKAR) was undertaken to simulate HCQ concentration-time profiles in plasma, whole blood, and lung in Chinese healthy populations. Since elderly patients have reduced glomerular filtration rate (GFR), simulations were conducted using a healthy Caucasian healthy population with renal injury (GFR 30 to 60 mL/min) and compared to a population with normal renal function to support the study design of prophylactic use of HCQ. In this analysis, 3 dosing regimens were simulated. Modeling suggests a loading dose of 400 mg for 3 days followed by 200 mg daily for 2 weeks should result in a shorter time to PK steady-state (Appendix 5). The 2-week treatment duration represents coverage for the duration of viral incubation time.

This protocol will investigate a single dosage of HCQ. Participants will receive a loading dose of 400 mg for 3 days followed by 200 mg daily for 11 days. Subsequent investigations will be encouraged to undertake a more rigorous exposure-response assessment to define optimal dosing, including exploration of the lowest possible effective dose, and possible alternate dosing schedules (i.e., weekly instead of daily).

3.2.5. *Rationale for Ascorbic Acid Control as a Comparator*

In healthy adults, COVID-19 infection is likely to express as a febrile disease with cough and fatigue, but variability in symptomatology (to include oligosymptomatic infection) is a possibility [28]. Study participants will have already been exposed to SARS-CoV-2, likely with quarantine as a result, so randomization is unlikely to affect future risk behavior with respect to SARS-CoV-2. Symptom reporting may vary based on participants' perception as to whether they are taking HCQ or ascorbic acid [29], but the primary study endpoints of infection and viral shedding are not affected.

Therefore, use of a control is acceptable and ethical both for participants' health and safety as well as ensuring the most rigorous trial design to evaluate PEP for SARS-CoV-2. Participants will be blinded to their allocation to the extent possible, with a comparator of ascorbic acid used to provide a similar taste as HCQ. Specifically, tablets will not be labeled as either HCQ or ascorbic acid, and an ascorbic acid tablet that is comparable in size and shape to HCQ will be used. All participants from the same household will be assigned the same regimen, so that comparisons cannot be made between the medications and to minimize crossover through drug sharing by household members.

The dose of ascorbic acid (500 mg for 3 days, then 250 mg daily for 11 days) is safe. All participants, regardless of assigned group, will be able to take additional ascorbic acid (e.g., over the counter vitamins, or through food) should they choose, as there is no known maximum daily safe dose of ascorbic acid. Clinical trial evidence has demonstrated that ascorbic acid, alone or in combination with other micronutrients, does not substantially reduce the risk of upper respiratory infections or severe consequences of infectious processes [30,31]; thus, ascorbic acid is not expected to have a prevention effect for SARS-CoV-19 and is considered a placebo-equivalent product for this study [32].

3.2.6. *Rationale for Enrolling the Index Case for the Index Case Sub-study*

The quantity of the virus shed by an individual frequently predicts transmissibility, and thus may relate to the likelihood of SARS-CoV-2 transmission. Index cases will be enrolled, and a nasal swab collected for SARS-CoV-2 testing to ascertain whether the index case was shedding virus and the level of viral shedding. The genotype of the virus shed will be investigated in a subset of participants. Evaluation of the paired samples (index case and contact) will provide evidence on whether the level of shedding is associated with SARS-CoV-2 transmission and whether subsequent infections among contacts were acquired within households.

3.2.7. *Rationale for Including Genetic Testing*

Genetic markers may be plausibly associated with SARS-CoV-2 acquisition. Recent studies indicate that ACE2 could be the host receptor for SARS-CoV-2. A number of ACE2 variants could reduce the association between ACE2 and SARS-CoV-2. Therefore, the expression of human ACE2 might be important for the susceptibility, symptoms, and outcome of SARS-CoV-2 infection [33]. The association between plausible genetic markers and SARS-CoV-2 infection and disease will be assessed in this study.

3.3. *Benefit/Risk Assessment*

COVID-19 can be unpredictable in its severity, but a 3.4% mortality rate has been observed among clinical pneumonia cases. The elderly (>60 years) and those with medical comorbidities are at highest risk of poor outcomes [1,2,3]. Moreover, transmission in younger persons amplifies infection in communities, putting susceptible persons at risk. There is no proven preventive drug other than transmission avoidance through barrier and droplet precautions, and in those likely exposed, quarantine for 14 days (beyond the viral incubation period) is currently recommended. HCQ has an excellent safety record at the proposed doses for 60 years and potent viral suppression *in vitro*. There is equipoise as to whether the *in vitro* efficacy of HCQ, or any other drug, will translate into preventive efficacy from disease or even a reduction in viral shedding. Thus, the potential benefit-to-risk ratio for testing HCQ as PEP is favorable in this population.

4. Objectives and Endpoints

Objectives	Endpoints
Primary	
• To test the efficacy of HCQ (400 mg orally daily for 3 days then 200 mg orally daily for an additional 11 days, to complete 14 days) to prevent incident SARS-CoV-2 infection, compared to ascorbic acid among contacts of persons with SARS-CoV-2 infection	• Polymerase chain reaction (PCR)-confirmed SARS-CoV-2 infection (self-collected samples collected daily Day 1 through Day 14)
Secondary	
• To determine the safety and tolerability of HCQ as SARS-CoV-2 PEP in adults	• Adverse events
• To test the efficacy of HCQ (400 mg orally daily for 3 days then 200 mg orally daily for an additional 11 days, to complete 14 days) to prevent incident SARS-CoV-2 infection 2 weeks after completing therapy, compared to ascorbic acid among contacts of persons with SARS-CoV-2 infection	• PCR-confirmed SARS-CoV-2 infection (self-collected samples collected daily Day 1 through Day 14 and collected 2 weeks after cessation of treatment [i.e., Day 28])
• To test the efficacy of HCQ to shorten the duration of SARS-CoV-2 shedding among those with SARS-CoV-2 infection in the HCQ PEP group	• SARS-CoV-2 viral shedding by PCR
• To test the efficacy of HCQ to prevent incident COVID-19	• PCR-confirmed COVID-19 diagnosis post start of HCQ therapy
Exploratory	
• *To conduct SARS-CoV-2 viral genotyping to assess the impact of HCQ on viral resistance to HCQ PEP*	• *Viral genotyping of SARS-CoV-2 in a subset of treated study participants who develop infection*
• To assess adherence to HCQ PEP	• Self-reported daily dosing *and study therapy concentration (if in sub-study)* in study participants

Objectives	Endpoints
• *To evaluate the pharmacokinetics (PK) of HCQ by infection status*	• *HCQ blood concentration*
• *To assess if there is an association between PK of HCQ and the efficacy and safety endpoints evaluated*	• *HCQ blood concentration, tolerability, and efficacy*
• *To evaluate if SARS-CoV-2 viral load in the index case is associated with SARS-CoV-2 infection in the contact*	• *SARS-CoV-2 semiquantitative PCR and genotyping*
• *To test the efficacy of HCQ to shorten the magnitude of SARS-CoV-2 shedding among those with SARS-CoV-2 infection in the HCQ PEP group*	• *Daily SARS-CoV-2 semiquantitative PCR*
• *To investigate the association of plausible genetic markers and susceptibility to SARS-CoV-2 infection and/or severity of COVID-19*	• *Rational gene candidates associated with SARS-CoV-2 infection and/or COVID-19 severity*

COVID-19: coronavirus disease; HCQ: hydroxychloroquine; PEP: post-exposure prophylaxis; SARS-CoV-2: severe acute respiratory syndrome coronavirus 2.

5. Study Design

5.1. *Overall Design*

The overarching goal of this study is to assess the effectiveness of HCQ PEP on the incidence of SARS-CoV-2 detection by polymerase chain reaction (PCR) to inform public health control strategies.

This is a randomized, multi-center, placebo-equivalent (ascorbic acid) controlled, blinded study of the efficacy of HCQ PEP for the prevention of SARS-CoV-2 infection in adults exposed to the virus.

This study will enroll up to 2000 asymptomatic men and women 18 to 80 years of age (inclusive) at baseline who are close contacts of persons with PCR-confirmed SARS-CoV-2 or clinically suspected COVID-19 and a pending SARS-CoV-2 PCR test. Eligible participants will be enrolled and randomized 1:1 to HCQ or ascorbic acid at the level of the household (all eligible participants in 1 household will receive the same intervention).

Participants will be counseled about the preliminary *in vitro* data on HCQ activity against SARS-CoV-2 and equipoise regarding efficacy in humans.

Participants may participate in a sub-study where they will be asked to provide a dried blood spot (DBS) sample for therapy concentration and PK of HCQ as well as for SARS-CoV-2 antibody testing, if possible.

Index cases will be invited to participate in a sub-study where they will be asked to provide a mid-nasal swab at baseline and complete questionnaires at baseline and Day 28 to assess the extent to which SARS-CoV-2 transmission occurs within household contacts and whether that is dependent on the quantity of the virus shed.

An independent data and safety monitoring board (DSMB) will be convened for this study with expertise in COVID-19 or respiratory viruses, PEP, and emerging epidemics; DSMB will include a biostatistician. The purpose of the DSMB is to monitor the study for operational futility, social harms, and efficacy.

This multi-center study will be conducted in high COVID-19 incidence areas in the United States. At the time of the writing of this document, the sites are expected to be western Washington (Snohomish, King, and Pierce counties) and New York, NY. As of 13 March 2020, there are 328 cases and 32 deaths in King County, most of them in Seattle. As of 13 March 2020, there are 158 cases in New York City. Both setting are urban areas; Seattle has a population of 3.5 million and New York has 8.6 million.

If additional data emerge on alternative effective agents for SARS-CoV-2 prophylaxis and/or data about HCQ as PEP, the protocol could be modified through an amendment to alter its sample size and evaluate an alternative therapy as PEP. Alternatively, this platform study could be modified through an adaptive design to test the efficacy of new potential agents for COVID-19 PEP.

5.2. *Participant and Study Completion*

Up to 2000 participants will be randomly assigned to study treatment. *Up to 2000 index cases will be enrolled.*

A participant is considered to have completed the study if he/she has completed all phases of the study including the last scheduled procedure shown in the Schedule of Activities (SoA).

This study will be considered completed when sufficient number of participants complete the study to enable appropriate evaluation of the primary endpoint.

6. Study Population

Prospective approval of protocol deviations to recruitment and enrollment criteria, also known as protocol waivers or exemptions, is not permitted.

6.1. *Close Contact Participants*

6.1.1. *Inclusion Criteria*

Participants are eligible to be included in the study only if all of the following criteria apply:

1. Men or women 18 to 80 years of age inclusive, at the time of signing the informed consent
2. Willing and able to provide informed consent
3. Had a close contact of a person (index) with known PCR-confirmed SARS-CoV-2 infection or index who is currently being assessed for COVID-19
   Close contact is defined as:
   a. Household contact (i.e., residing with the index case in the 14 days prior to index diagnosis or prolonged exposure within a residence/vehicle/enclosed space without maintaining social distance)
   b. Medical staff, first responders, or other care persons who cared for the index case without personal protection (mask and gloves)
4. Less than 4 days since last exposure (close contact with a person with SARS-CoV-2 infection) to the index case
5. Access to device and internet for Telehealth visits
6. Not planning to take HCQ in addition to the study medication

6.1.2. *Exclusion Criteria*

Participants are excluded from the study if any of the following criteria apply:

1. Known hypersensitivity to HCQ or other 4-aminoquinoline compounds
2. Currently hospitalized
3. Symptomatic with subjective fever, cough, or shortness of breath
4. Current medications exclude concomitant use of HCQ
5. Concomitant use of other anti-malarial treatment or chemoprophylaxis, including chloroquine, mefloquine, artemether, or lumefantrine.
6. History of retinopathy of any etiology
7. Psoriasis
8. Porphyria
9. Known bone marrow disorders with significant neutropenia (polymorphonuclear leukocytes <1500) or thrombocytopenia (<100 K)
10. Concomitant use of digoxin, cyclosporin, cimetidine, amiodarone, or tamoxifen
11. Known moderate or severe liver disease
12. Known long QT syndrome
13. Severe renal impairment

14. Use of any investigational or non-registered drug or vaccine within 30 days preceding the first dose of the study drugs or planned use during the study period

6.2. *Index Cases*

6.2.1. *Inclusion Criteria*

1. *Men or women ≥18 years of age inclusive, at the time of signing the informed consent*
2. *Willing and able to provide informed consent*
3. *Currently diagnosed with known PCR-confirmed SARS-CoV-2 infection or is currently being assessed for COVID-19*
4. *A close contact is enrolled in the study*

6.2.2. *Exclusion Criterion*

1. *Currently hospitalized*

6.3. *Screen Failures*

Screen failures are defined as participants who consent to participate in the clinical study but are not subsequently randomly assigned to study treatment. A minimal set of screen failure information is required to ensure transparent reporting of screen failure participants to meet the Consolidated Standards of Reporting Trials publishing requirements and to respond to queries from regulatory authorities. Minimal information includes demography, screen failure details, eligibility criteria, and any serious adverse event (SAE).

Individuals who do not meet the criteria for participation in this study (screen failure) can be rescreened if there is a change in their eligibility.

6.4. *Recruitment*

Each site will establish local recruitment and screening methods that operationalize protocol-specified requirements for eligibility determination in a manner that is tailored to and most efficient for the local study setting and target study population. Each site will use a variety of recruitment approaches, including direct recruitment at clinics, referrals from other providers and SARS-CoV-2 testing sites and laboratories, and use of online and social networking websites and apps. Recruitment materials will educate participants about COVID-19, transmission within households, epidemiology in the community, and the *in vitro* data on HCQ limiting SARS-CoV-2 cell entry.

Initially, the study will enroll from 2 clinical sites: western Washington and New York, which have established track records of high quality clinical research integrated into clinical care settings; annual retention rates in clinical trials conducted in these sites exceed 80% to 90%. The sites have large COVID-19 epidemics with regulation limiting contact to reduce infectious spread.

6.5. *Co-enrollment Guidelines*

Close contact participants may be co-enrolled in other research studies, provided that these are observational studies only. Any other exception requires approval of the Co-Principal

Investigators. The study team should be consulted for co-enrollment in studies that do not meet this guidance or if there are questions about eligibility for co-enrollment. For any co-enrolled study, combined blood draws should not exceed current Red Cross phlebotomy guidance.

Index case participants may enroll at any point in another study.

7. Treatments

Study treatment is defined as any investigational treatment(s), marketed product(s), placebo, or medical device(s) intended to be administered to a study participant according to the study protocol.

7.1. *Treatments Administered*

**Study Treatment Name**	Hydroxychloroquine sulfate	Ascorbic acid
**Dosage Formulation**	200 mg (155 mg base) tablets	250 mg tablets
**Route of Administration**	Oral	
**Dosing Instructions**	Take 2 tablets for the first 3 days and 1 tablet for the subsequent 11 days, for a total of 14 days of treatment. Take at approximately the same time of the day with a meal or a glass of milk. If a dose is missed, it should be taken as soon as possible. If it is less than 4 hours before the next dose, the dose should be skipped.	
**Packaging and Labeling**	The medication for home delivery will be dispensed in an unmarked container with the study label. The container will be labeled with a unique identifier. The container will be packed in a standard box used for mail delivery of medications as needed.	
**Manufacturer**	To be determined	To be determined

7.2. *Risks to the Participants*

7.2.1. *Risks Associated with PEP Administration with HCQ*

The risks associated with PEP reflect the adverse events (AEs) related to HCQ administration. With tens of millions of doses administrated for malaria and autoimmune diseases, the side effect profile of HCQ is well described and the drug is generally well tolerated. With short-term administration (as opposed to chronic/year-long use in rheumatologic disease management), the major AEs are gastrointestinal (nausea, vomiting, dyspepsia, abdominal cramps, and diarrhea) and transient skin rashes. The gastrointestinal symptoms may vary by specific generic manufacturer of HCQ [35] and are best managed by taking the drug with food or a glass of milk (Appendix 6). A transient rash, most commonly morbilliform or psoriasiform, can develop in 10% of participants, often with a sustained loading dose, and is often managed by lowering the dose. To avoid this potential side effect, this protocol is using a short loading dose, not a sustained one. Uncommonly, idiosyncratic leukopenia/thrombocytopenia can occur and the drug

should not be given to those with underlying bone marrow disorders. Lastly, hypoglycemia can occur and those taking insulin or glucose-lowering drugs are at risk; blood glucose should be monitored.

Minor blurry vision may occur with acute HCQ use, is reversible, dose-related, and is generally not severe. It may also be associated with mild headaches. It is caused by deposition of HCQ in the cornea or ocular muscle weakness, and is not a predictor of long-term toxicity. Participants experiencing minor blurry vision may adjust to it with counselling and reassurance that it is reversible and not progressive.

More severe rashes, including drug-related eosinophilia with systemic symptoms and Stevens-Johnson syndrome occur rarely.

Long-term manifestations of HCQ, including retinitis, renal and hepatic disease, and cardiomyopathy (Appendix 4), are not likely in this 2-week PEP exposure.

7.2.2. *Risks Associated with COVID-19 Surveillance and Quarantine*

Enrollment in this protocol will not impact the public health department's advice for self-quarantine. That said, quarantine is known to be stressful, especially for healthcare workers. Enrollment may improve morale during public health quarantine. Since there is clinical equipoise regarding HCQ efficacy on viral shedding, participants will be counseled to follow recommendations for self-quarantine.

7.3. *Strategies to Minimize Risk*

7.3.1. *Dose Selection*

The recommended dose of HCQ for chronic use in lupus erythematosus is 200 mg to 400 mg daily—a dose that is safely taken for years including among elderly patients (≥65 years). The selection of 400 mg for 3 days then 200 mg daily for 11 days is likely to be safe with transient AEs (gastrointestinal symptoms and rashes) that are self-remitting.

7.3.2. *Management of Participants to Limit Risks of SARS-CoV-2 Transmission*

To limit the transmission of SARS-CoV-2, participants will receive visits via secure Telehealth in order to limit the movement of persons with potential SARS-CoV-2 and leave clinical space free for patients seeking care. Also, to limit exposure in waiting rooms and pharmacies, clinical specimens will be self-collected and medications delivered to homes.

7.4. *Dose Modification and Toxicity Management*

If a study therapy dose is missed, it should be taken as soon as possible. If it is less than 4 hours before the next dose, the dose should be skipped.

Modification for toxicities is discussed below. Only toxicities related to study medications provided through the study will be considered in the toxicity management section.

Grade 1 or 2

Participants who develop Grade 1 or 2 toxicity (per division of acquired immunodeficiency syndrome [DAIDS] adverse event [AE] Grading Table; see: https://rsc.tech-

res.com/docs/default-source/safety/division-of-aids-(daids)-table-for-grading-the-severity-of-adult-and-pediatric-adverse-events-corrected-v-2-1.pdf) that is considered to be related to study medication may continue study treatment at the discretion of the site Investigator with close follow-up. If a participant chooses to discontinue study treatment, the site should notify the study protocol team within 7 days. These participants will remain on study, off study treatment, and have all evaluations performed.

Grade 3

- Participants who develop a Grade 3 symptomatic toxicity thought by the site Investigator to be related to study drug should have HCQ PEP withheld, and the site should consult with the Core Protocol team. The participant should be reevaluated every 2 days until the AE returns to Grade ≤2, at which time study drug may be reintroduced at the discretion of the site Investigator in consultation with the protocol team.
- Participants experiencing Grade 3 toxicity requiring permanent discontinuation of study medication should be followed up weekly until resolution of the toxicity. Participants will have premature study treatment discontinuation evaluations performed. These participants will remain on study, off study treatment, and have all evaluations performed per the SoA.

Grade 4

- Participants who develop a Grade 4 symptomatic toxicity will have study medication permanently discontinued, and the site should notify study team within 48 hours.
- Participants experiencing Grade 4 toxicity requiring permanent discontinuation of HCQ PEP should be followed up weekly until resolution of the AE or return to baseline. These participants will remain on study, off study treatment, and have all evaluations performed per the SoA.

Specific Management of Toxicities Related to Study-Provided Drugs

Gastrointestinal disturbance

Gastrointestinal disturbance (nausea, vomiting, diarrhea) is a common known possible side effect of HCQ. Dividing doses by 6 hours and taking with food or milk may improve tolerability.

Visual disturbances

Suspected visual changes should be evaluated for possible etiologies. Participants experiencing Grade 1 or Grade 2 vision changes may continue use, at the discretion of the site Investigator. Participants who develop a Grade 3 symptomatic toxicity thought by the site Investigator to be related to study drug should have HCQ PEP withheld, and the site should consult with the Core Protocol team. The participant should be reevaluated every 2 days until the AE returns to Grade ≤2, at which time study drug may be reintroduced at the discretion of the site Investigator in consultation with the protocol team.

Allergic reactions

HCQ should be discontinued permanently if a serious allergic reaction is suspected. These participants will remain on study, off study treatment, and have all evaluations performed per the standard operating procedure(s).

7.5. *Method of Treatment Assignment*

Participants will be randomized in a 1:1 ratio to HCQ or ascorbic acid at the level of the household (all eligible participants in 1 household will receive the same intervention). The randomization plan will be written by the Study Statistician.

The randomization code and resulting allocation list will be generated and maintained by the Study Statistician. The list will be blocked and stratified by site and contact type (household versus healthcare worker).

7.6. *Blinding*

This is a blinded study. Eligible participants will receive one of the following therapies:

- HCQ 400 mg orally daily for 3 days then 200 mg orally daily for an additional 11 days
- Ascorbic acid 500 mg orally daily for 3 days then 250 mg orally daily for 11 days

HCQ and ascorbic acid will appear similar, and taste will be partially masked as HCQ can be bitter and ascorbic acid will be sour.

The participants will be blinded to their randomization group once assigned. Within 24 to 48 hours of enrollment, the unblinded Study Pharmacist will use the randomization code revealed at the point of randomization to provide the participant with their group assignment and dispense the allocated study medication in a bottle marked with the study label. The medication and medication information, mid-nasal swabs sufficient to complete the study procedures, *DBS sampling kit, if within the sub-study,* and study instructions will be delivered to the participant.

7.7. *Preparation/Handling/Storage/Accountability*

Drugs should be stored at room temperature, as per package insert. Records must be maintained that document receipt, release for dosing, disposal, or return to the sponsor.

7.8. *Treatment Compliance*

The participant will be contacted to ensure that they received the box of study supplies; were able to collect the first mid-nasal swab and store it appropriately; and took their first day of medication. Participants will be asked to complete a Survey that includes information regarding treatment administration. *In a sub-study, HCQ concentration via a DBS will also be evaluated.*

Consultation via Telehealth, text messaging, or telephone will be available to provide support to the participant to complete study procedures.

7.9. *Concomitant Therapy*

Participants will be asked about concomitant medications at the screening/baseline evaluation visit. During the study, participants will be asked to complete Surveys (Daily Survey and Exit Contact Survey) that include information regarding any medication or vaccine (including over-the-counter or prescription medicines, vitamins, and/or herbal supplements) that the participant receives during the study. At each contact, the Investigator or designee should question the participant about any medication taken.

7.9.1. *Prohibited Medications*

Prohibited medications include digoxin, cyclosporin, cimetidine, amiodarone, tamoxifen, and antimalarial medications (including chloroquine, artemether, lumefantrine, mefloquine).

7.9.2. *Precautionary Medications*

Use of medications classified as precautionary is not study prohibitory but will be discussed with the study clinician and the participant. These medications include androgens, antidiabetic agents, phenothiazines, beta-blockers, cardiac glycosides, citalopram, dapsone, escitalopram, haloperidol, herbs, maitake, monoamine oxidase inhibitors, pegvisomant, prothionamide, quinolones, salicylates, and selective serotonin reuptake inhibitors.

7.10. *Treatment After the End of the Study*

No additional treatment will be provided at the end of the study.

8. Discontinuation/Withdrawal Criteria

8.1. *Discontinuation of Study Treatment*

Study treatment will be discontinued for the following reasons:

- Requirement for prohibited concomitant medications or other contraindication to HCQ
- Occurrence of an AE requiring discontinuation of study medication
- Request by participant to terminate study treatment
- Clinical reasons believed to be life-threatening by the physician, even if not addressed in Section 7.2

Participants who stop study treatment should continue study participation off study medication with continued evaluations as per the SoA. The reason for study medication discontinuation should be recorded.

Participants who are identified as SARS-CoV-2 positive by PCR at baseline should continue study therapy and study participation.

Participants with a clinical diagnosis of COVID-19 should continue on study therapy unless they are started on COVID-19 treatment outside of the study.

8.2. *Withdrawal from the Study*

- A participant may withdraw from the study at any time at his/her own request or may be withdrawn at any time for the following reasons:
  - At the request of the primary care provider if he/she thinks the study is no longer in the best interest of the participant
  - Participant is judged by the Investigator to be at significant risk of failing to comply with the provisions of the protocol as to cause harm to self or seriously interfere with the validity of the study results
  - At the discretion of the Institutional Review Board/Ethics Committee or government agencies as part of their duties, Investigator, or industry supporter
- If the participant withdraws consent for disclosure of future information, the sponsor may retain and continue to use any data collected before such withdrawal of consent.
- If a participant withdraws from the study, he/she may request destruction of any samples taken and not tested, and the Investigator must document this in the site study records.
- See SoA for data to be collected at the time of study discontinuation and follow-up and for any further evaluations that need to be completed.

8.3. *Lost to Follow-up*

A participant will be considered lost to follow-up if he/she is unable to be contacted by the study site.

The following actions must be taken if a participant fails to comply with required study procedures:

- The site must attempt to contact the participant as soon as possible and counsel the participant on the importance of maintaining the assigned procedure schedule and ascertain whether or not the participant wishes to and/or should continue in the study.
- Before a participant is deemed lost to follow-up, the Investigator or designee must make every effort to regain contact with the participant (where possible, 3 telephone calls and, if necessary, a certified letter to the participant's last known mailing address or local equivalent methods). These contact attempts should be documented in the participant's medical record.
- Should the participant continue to be unreachable, he/she will be considered to have withdrawn from the study with a primary reason of lost to follow-up.

9. Study Encounters

The current COVID-19 pandemic has placed a significant burden on the healthcare system. For this study, specimen and data collection will be conducted to minimize impact of non-ill participants within the healthcare system. If the participant is assessed as eligible, contact between study participants and study personnel will occur via a Health Insurance Portability and Accountability Act (HIPAA)-compliant video conference (Telehealth), although in-person visits either at the participant's home or at a research center are permitted.

Participants will be instructed to seek clinical care should they manifest any signs or symptoms of COVID-19.

9.1. *Close Contact Participants*

9.1.1. *Screening/Baseline Evaluation: Day 0/1*

Participants will be assessed for study eligibility through a screening conducted through a web-based screening tool, HIPAA-compliant video conference (Telehealth), telephone, or text messaging.

Day 0 evaluations are as follows:

- Informed consent
- Collection of demographic information
- Collection of past and current medical conditions, including known pregnancy and/or lactation status
- Collection of concomitant medication information
- Collection of information regarding exposure to the index case
- Check of inclusion and exclusion criteria

Eligible participants will be randomized with all members of a household assigned to the same arm. Participants will receive a swab kit either via courier or mail, which includes a Quick Start Instruction Card, swabs, plastic tubes for swab collection, and a return box with affixed Category B UN3373 label, as required by International Air Transport Association (IATA) guidelines [36].

The participant will do the following on Day 1:

- Collect mid-nasal swab for PCR
- Take study therapy (HCQ or ascorbic acid, as assigned)
- Complete Daily Survey (online, Telehealth, telephone, or text messaging). This Survey will include confirmation of adherence to study therapy dosing and daily swab collection, concomitant medication review, and symptoms review.
- *If in DBS Sub-study, collect DBS samples for analysis of anti-SARS-CoV-2 antibodies (if possible).*

Instructions for skin puncture and DBS sample preparation are provided in Appendix 6. A study team member will be available via Telehealth, telephone, or text messaging to provide support for completion of this study procedure.

Screening/Day 0 and Day 1 procedures may occur on the same day.

The index case SARS-CoV-2 positive test results will be confirmed through self-report of the contact participant. *If in the index case sub-study, shedding of SARS-CoV-2 will be assessed by PCR of a mid-nasal swab (Section 9.2).*

9.1.2. *Day 2 Through Day 13*

The participant will do the following every day from Day 1 through Day 13, inclusive:

- Collect mid-nasal swab for PCR
- Take study therapy (HCQ or ascorbic acid, as assigned)
- Complete Daily Survey (online, telephone, or text messaging). This Survey will include confirmation of adherence to study therapy dosing and daily swab collection, concomitant medication review, and symptoms review.
- *If in DBS Sub-study, collect DBS samples for analysis of HCQ concentration at any time during this period (1 to 5 times) after study drug dosing has commenced. No more than 1 sample per day should be collected.*

A study team member will be available via Telehealth, text messaging, or telephone to provide support for completion of study procedures. The courier will collect the swabs at minimum within 1 or 2 days of Day 7 and Day 13 and potentially as frequently as daily.

9.1.3. *Day 14*

The participant will do the following on Day 14:

- Collect mid-nasal swab for PCR
- Take study therapy (HCQ or ascorbic acid, as assigned)
- Complete Daily Survey (online, telephone, or text messaging). This Survey will include confirmation of adherence to study therapy dosing and daily swab collection, concomitant medication review, and symptoms review.
- *If in DBS Sub-study, collect DBS samples for analysis of HCQ concentration, if not already collected.*

A study team member will be available via Telehealth, text messaging, or telephone to provide support for completion of study procedures.

Participants are instructed to place their self-collected nasal swabs directly into the plastic tube that is pre-labeled with a unique barcode. Next, participants are instructed to place the plastic tube containing the self-collected nasal swab into a specimen bag, pre-packaged with an absorbent sheet, and then place the specimen bag into the provided return shipping box. Category B UN3373 stickers are affixed to the outside of the return box. Previous testing has demonstrated that respiratory viral ribonucleic acid (RNA) is stable in room temperature for up to 1 week. The courier will collect the swabs at minimum within 1 or 2 days of Day 14.

9.1.4. *Exit Contact (Day 28±3)*

The Exit Contact will be conducted through Telehealth, online, by telephone, or by text messaging.

Exit Contact evaluations are as follows:

- Collect mid-nasal swab for PCR
- Review AEs

- Complete Exit Contact Survey (online, Telehealth, telephone, or text messaging). This Survey will include concomitant medication review and symptoms review.
- The Exit Contact Survey will include evaluation for SARS-CoV-2 infection and/or COVID-19 and associated testing and medical care.
- *If in DBS Sub-study, collect DBS samples for analysis of anti-SARS-CoV-2 antibodies (if possible).*

Clinical outcomes will be confirmed through the electronic health record, if possible.

The final swab will be collected by the courier or returned via the postal service.

9.1.5. *Participants who Develop COVID-19 During the Study*

Participants who develop symptoms concerning for COVID-19 will be instructed to contact their provider for clinical care and testing. Study results will be available to participants after study completion as they are not tested in real-time for clinical diagnosis.

Participants who develop SARS-CoV-2 infection may be sent a home pulse oximeter to measure the oxygen saturation to provide direct information for clinical assessment.

Participants with a clinical diagnosis of COVID-19 should continue on study therapy unless they are started on COVID-19 treatment outside this study. Other study procedures will continue, with early exit possible to document COVID-19 diagnosis.

9.2. *Index Cases*

9.2.1. *Baseline Evaluation: Day 1*

Participants will be assessed for study eligibility through a screening conducted through a web-based screening tool, Telehealth, telephone, or text messaging.

Day 1 evaluations are as follows:

- *Informed consent*
- *Collection of demographic information*
- *Check of inclusion and exclusion criteria*

Eligible participants will receive a swab kit either via courier or mail, which includes a Quick Start Instruction Card, a swab, a plastic tube for swab collection, and a return container with affixed Category B UN3373 label, as required by International Air Transport Association (IATA) guidelines [36].

The participant will do the following on Day 1:

- *Collect mid-nasal swab for PCR*
- *Complete Survey (online, Telehealth, telephone, or text messaging). This Survey will include assessment of risk for COVID-19 acquisition, engagement with testing and treatment, and symptom review.*

9.2.2. *Exit Contact (Day 28)*

The Exit Contact will be conducted through Telehealth, online, by telephone, or by text messaging.

Exit Contact evaluations are as follows:

- *Complete Exit Contact Survey (online, Telehealth, telephone, or text messaging). This Survey will include assessment of risk for COVID-19 acquisition, engagement with testing and treatment, and symptom review.*

Clinical outcomes will be confirmed through the electronic health record, if possible.

9.3. *Participant Reimbursement*

Participants will be reimbursed on Day 14 and Day 28. *Index cases enrolled in the index case sub-study will receive a reimbursement on Day 28.* No reimbursement will be provided to index cases for referral of their close contacts. No reimbursement will be provided for unscheduled Telehealth visits requested by the participants for support with study procedures.

10. Study Assessments and Procedures

- Study procedures and their timing are summarized in the SoA.
- Protocol waivers or exemptions are not allowed.
- Immediate safety concerns should be discussed with the sponsor immediately upon occurrence or awareness to determine if the participant should continue or discontinue study treatment.
- Adherence to the study design requirements, including those specified in the SoA, is essential and required for study conduct.
- All baseline evaluations must be completed and reviewed to confirm that potential participants meet all eligibility criteria. The Investigator will maintain a screening log to record details of all participants screened and to confirm eligibility or record reasons for screening failure, as applicable.
- Procedures conducted as part of the participant’s routine clinical management and obtained before signing of the informed consent form may be utilized for screening or baseline purposes provided the procedures met the protocol-specified criteria and were performed within the time frame defined in the SoA.
- *Blood samples will only be collected as a part of a sub-study. The maximum amount of blood collected from each participant over the duration of the study, including any extra assessments that may be required, will not exceed 10 mL.*

10.1. *Efficacy Assessments*

10.1.1. *Mid-nasal Swab*

Participants will collect daily mid-nasal swabs for the initial 14 days and again at the Exit Contact for viral detection.

Index case participants in the index case sub-study will submit their baseline nasal swab for SARS-CoV-2 testing by PCR.

Participants will receive a swab kit either via courier or mail, which includes a Quick Start Instruction Card, swabs, plastic tubes for swab collection, and a return box with affixed Category B UN3373 label, as required by IATA guidelines [36].

Participants will collect and store their nasal swabs in a box for return.

The used swabs will be collected by the courier or returned via the postal service.

Swabs will be subjected to RNA amplification and tested for SARS-CoV-2, as applicable.

10.1.2. *Participant Survey*

Participants will be asked to complete Surveys (Daily Survey and Exit Contact Survey) that will include questions about symptoms from both the drug regimen and virus infection, review of concomitant medications, and other pertinent topics include information on any symptoms that may be associated with COVID-19.

10.2. *Adverse Events*

AE information will only be collected on close contact participants. For information regarding AEs in index case participants see Section 10.2.7.

Participants will be asked to complete Surveys (Daily Survey and Exit Contact Survey) that include information on any symptoms that they are experiencing. In addition, AE review by a staff member (via telephone, Telehealth, or text messaging) will be performed.

All AEs must be recorded on electronic case report forms (eCRFs) if any of the following criteria have been met:

- All AEs meeting SAE definition
- All AEs judged by participant or clinician to be associated with study medication

10.2.1. *Serious Adverse Events*

An SAE is defined as any untoward medical occurrence that:

- Results in death
- Is life-threatening
- Requires inpatient hospitalization or prolongation of existing hospitalization
- Results in persistent or significant disability/incapacity
- Is an important medical event that may not be immediately life-threatening or result in death or hospitalization but may jeopardize the patient or may require intervention to prevent one of the other outcomes listed in the definition above

All AEs that are recorded will have their severity graded. To grade AEs, sites should refer to the DAIDS AE Grading Table, corrected Version 2.1, July 2017, which can be found on the DAIDS Regulatory Support Center website at https://rsc.tech-res.com/docs/default-source/safety/division-of-aids-(daids)-table-for-grading-the-severity-of-adult-and-pediatric-adverse-events-corrected-v-2-1.pdf.

10.2.2. *AE and SAE Attribution to Study Medication*

All AEs and SAEs should have attribution recorded as related or not related to study medication, in the judgment of the site Investigator.

10.2.3. *AE and SAE Reporting*

- ***SAEs will be reported within 48 hours to the Sponsor.***
- ***Grade 3 and 4 AEs assessed as related to the study medication by the clinician will be reported to the Sponsor within 48 hours.***
- ***Grade 1 and 2 AEs assessed as related to the study medication by the clinician will be reported to the Sponsor within 2 weeks of the participant's Exit Contact.***

10.2.4. *Reporting of Use During Pregnancy*

- Medication exposure during pregnancy will be reported to the Sponsor within 2 weeks of *the participant's Exit Contact*.

10.2.5. *Reporting of Misuse of Drug*

- Misuse and abuse of medication outside the protocol will be reported to the Sponsor within 2 weeks of *the participant's Exit Contact.*

10.2.6. *Treatment of Overdose*

Overdose of HCQ should be managed according to the labeling information (see Appendix 4).

Ascorbic acid exhibits low toxicity; risks from overdose are expected to be minimal.

Overdose of study medication should be reported to the Sponsor within 48 hours.

10.2.7. *AEs Among Index Case Participants*

AEs among the index case participants in the index case sub-study will not be collected or reported as index cases are not receiving study medication or interventions.

10.3. *Safety and AE Assessments*

Safety will be assessed via participant Surveys, as shown in the SoA.

Participants will be asked to complete Surveys (Daily Survey and Exit Contact Survey) that include questions about their health, healthcare seeking, symptoms, illness within their household, contact, and mobility. Qualifying events will be recorded on the eCRF and reported as AEs, as described in Section 10.2.

Social harms will be assessed throughout the study, reported using the Social Harms form, and reported to the Sponsor within 48 hours.

10.4. *Dried Blood Spot Optional Sub-study*

Up to 1800 participants will be enrolled in the DBS Sub-study. DBS will be requested, but not required, of all participants. For those study sites not wishing to participate in the DBS Sub-study, this will not be considered a protocol deviation.

Participants will receive instructions for DBS self-collection in writing, with telephone, Telehealth, and text messaging options as support. Once cards have been dried, they will be collected by the study courier and returned to the laboratory.

The aim of the DBS Sub-study is to evaluate HCQ drug concentrations as an adherence measure and the PK of HCQ. If serological assays for SARS-CoV-2 are available, DBS may be tested for SARS-CoV-2 antibodies.

Pharmacokinetics

The exposure-response relationship of HCQ in the PEP of participants exposed to SARS-CoV-2 has not been established. Population PK analyses can be used to further inform dose selection in other populations and support concentration-response investigations with efficacy and safety outcomes.

To accomplish this, sparse PK sampling techniques can be employed. This would involve collection of whole blood at 1 to 5 times after dosing has commenced. The time of collection post-dose can be random; however, no more than 1 sample per day should be collected.

Given the long half-life of HCQ, samples for several weeks after the last dose are also informative to the population PK model development.

The basic requirements for PK sampling are as follows:

1. *Accurate record of time of the dose prior to the blood sampling (dd:mm:yy; hh:mm)*
2. *Accurate recording of time of blood sampling (dd:mm:yy; hh:mm) for each blood sampling.*
3. *Whole blood can be obtained by venipuncture or capillary blood by skin puncture using a lancet.*
4. *Approximately 100 µL of blood is then applied to filter paper as outlined in Appendix 6.*

Anti-SARS-CoV-2 Antibody Testing

DBS sample for serology will be collected at Day 1 and Day 28 and tested for SARS-CoV-2 antibodies provided that an appropriate test is available.

10.5. *Genetic Testing*

10.5.1. *Samples Used for Genetic Testing*

Dried blood spot and mid-nasal swab samples collected in the study could be used potentially for genetic studies. During the consent process, participants can opt out of genetic testing.

10.5.2. *Genetic Testing Procedures*

Studies conducted on stored samples will investigate genes that may play a role in susceptibility to SARS-CoV-2 infection or other respiratory viruses and/or severity of COVID-19. The samples will not be used to study genes related to diseases other than SARS-CoV-2 or other respiratory viruses.

10.5.3. *Genetic Information That Will Be Obtained*

Samples will be tested for the presence of genetic markers, which the investigators believe, on the basis of available medical literature, to possibly be rationally associated with susceptibility to SARS-CoV-2 infection and/or the severity of COVID-19. Genome-wide screening (such as "SNP screening") is not planned.

10.6. *Biohazard Containment*

As the transmission of SARS-CoV-2 and other respiratory droplet pathogens can occur through contact with respiratory droplets and contaminated surfaces, precautions will be employed by all personnel in the handling of all specimens for this study, as currently recommended by the Centers for Disease Control and Prevention and the National Institutes of Health.

All dangerous goods and materials, including diagnostic specimens and infectious substances, must be transported using packaging mandated by Code of Federal Regulations 42 Part 72. Please refer to instructions detailed in the IATA Dangerous Goods Regulations.

11. Statistical Considerations

11.1. *Sample Size Determination*

Not all individuals exposed to COVID-19 will become infected, and early estimates place family members at a 3% to 15% risk of infection after likely exposure [32, 34]. The sample size required to show a 50% protective efficacy of PEP is entirely dependent on that attack rate. Sample size calculations used an estimated 6% attack rate.

A sample size will be chosen for each group (HCQ and ascorbic acid) to achieve 80% power with 0.05 two-sided Type 1 error for the endpoint of any laboratory-detected incident SARS-CoV-2, exclusion of participants with positive SARS-CoV-2 at baseline, an attack rate of 6%, 50% efficacy of HCQ in reducing SARS-CoV-2 incidence, and randomizing the first household member enrolled and subsequent household members to the same intervention. With these assumptions, a cohort of 1498 households (749 in the control and 749 in the intervention for each cohort) will provide at least 80% power to detect a decrease in SARS-CoV-2 incidence from 6% to 3%. As household size increases, power will increase.

Household members will be randomized as a unit but analyzed as individuals.

The table below estimates power assuming that households are randomized until either 1498 households are randomized or 1498 individuals are randomized. Each row shows the distribution of the numbers of individuals within a household who are randomized. Individual risk is assumed to be independent. Power may be lower depending on correlation in risk within and between households (Table 1).

Table 1 Power to Detect a Decrease from 6% to 3% Under Hypothetical Distributions of Number of Household Contacts

	Distribution of Number Randomized Within Household					Number of Households is the Target			Number of Participants is the Target		
**Number in Household**	**1**	**2**	**3**	**4**	**5**	N_{HH}	N_p	**Power**	N_{HH}	N_p	**Power**
A	0.8	0.1	0.06	0.03	0.01	1498	2022	89%	1108	1498	78%
B	0.75	0.15	0.06	0.03	0.01	1498	2097	90%	1070	1498	78%
C	0.25	0.35	0.16	0.13	0.11	1498	3745	99%	600	1498	76%

N_{HH}: number of households; Np: number of participants.

These power calculations indicate that final power will depend heavily on the population enrolled. To mitigate the potential loss in power from lack of information about cluster size, the final target sample size will be estimated midway through the study based on the observed distribution of the number of household contacts. The procedure for redetermining sample size will be described in an Interim Monitoring Plan approved by the DSMB.

11.2. *Populations for Analyses*

For analysis purposes, the following populations are defined:

Population	Description
Intention to Treat	All enrolled participants who are contacts
Modified Intention to Treat (MITT)	Contacts who are SARS-CoV-2 negative by PCR at the baseline visit and whose corresponding index case is SARS-CoV-2 positive by report or PCR
PK evaluable	Participants from the DBS Sub-study with at least 1 interpretable PK sample

DBS: dried blood spot; PK: pharmacokinetic; PCR: polymerase chain reaction; SARS-CoV-2: severe acute respiratory syndrome coronavirus 2.

Note: All PCR negative household contacts will be included in the MITT analyses.

11.3. *Statistical Analyses*

The Statistical Analysis Plan will be developed and finalized before database lock and will describe the participant populations to be included in the analyses, and procedures for accounting for missing, unused, and spurious data. This section is a summary of the planned statistical analyses of the primary and secondary endpoints (these are listed in Section 4).

11.3.1. *Efficacy Analyses*

Demographic characteristics (age, sex, race) of each study group will be tabulated.

The mean age (plus range and standard deviation) by sex of the enrolled participants, as a whole and per group, will be calculated.

The primary analysis will be performed using the modified Intention-to-Treat population. If the participant is not SARS-CoV-2 positive by laboratory test, their infection time will be censored at the last negative swab test date at or before the Day 14 swab. A logistic model stratified by site and accounting for within-household correlation will be used to assess the efficacy of the HCQ group compared to control. To aid understanding of the results from the logistic model, cumulative incidence Kaplan-Meier curves of time to infection will be presented by site and treatment arm.

Subgroup Analyses

All subgroup analyses will be pre-specified in the Statistical Analysis Plan. Any further subgroups will be considered *ad hoc*.

Missing Data

Due to the design of the study and retention activities, we expect to be able to categorize all infections as either incident or prevalent at baseline. However, in the unlikely event of a missing test result, the missing data will be imputed.

Additional analyses will be described in the Statistical Analysis Plan finalized before database lock.

11.3.2. *Safety Analyses*

All safety analyses will be performed on the Intention-to-Treat population. AEs and social harms will be compared by study group.

11.3.3. *Pharmacokinetic Analysis*

Sparse PK from DBS will be analyzed using standard population PK analysis methodologies using standard software such as NONMEM® V7.4 or Phoenix NLME V8.2.

11.3.4. *Exploratory Exposure-Response Analyses*

PK-evaluable participants will have post-hoc individual concentration profiles and exposure estimates determined for exploratory exposure-response analyses against primary and secondary efficacy and safety endpoints. Exploratory PK/pharmacodynamic analyses will be performed as the data allow.

11.3.5. *Exploratory Transmission Analyses*

The index case sub-study will assess the risk of SARS-CoV-2 transmission and the association with the semi-quantitative PCR results. Also, the risk of transmission between close contacts will be assessed by determining whether the viruses are a genetic match among household members through SARS-CoV-2 sequencing.

11.3.6. *Combined Study Analysis*

This protocol is being published as a model protocol for other institutions to consider as they undertake studying PEP of SARS-CoV-2 infection. It is hoped that individual patient data from similar studies can be pooled into a combined study analysis. De-identified data from the present study will be made available for these purposes in accordance with the funder's open access policy (https://www.gatesfoundation.org/how-we-work/general-information/open-access-policy).

12. References

1. Huang C, Wang Y, Li X, et al. Clinical features of patients infected with 2019 novel coronavirus in Wuhan, China. Lancet. 2020 Feb 15;395(10223):497-506. doi: 10.1016/S0140-6736(20)30183-5. Epub 2020 Jan 24.
2. Zhu N, Zhang D, Wang W, et al. A novel coronavirus from patients with pneumonia in China, 2019. N Engl J Med. 2020 Feb 20;382(8):727-733. doi: 10.1056/NEJMoa2001017. Epub 2020 Jan 24.
3. Chen N, Zhou M, Dong X, et al. Epidemiological and clinical characteristics of 99 cases of 2019 novel coronavirus pneumonia in Wuhan, China: a descriptive study. Lancet. 2020 Feb 15;395(10223):507-513. doi: 10.1016/S0140-6736(20)30211-7. Epub 2020 Jan 30.
4. WHO News Release. Statement on the second meeting of the International Health Regulations (2005) Emergency Committee regarding the outbreak of novel coronavirus (2019-nCoV). 2020 Jan 30.
5. White House Press Release. https://www.whitehouse.gov/presidential-actions/proclamation-declaring-national-emergency-concerning-novel-coronavirus-disease-covid-19-outbreak. 2020 Mar 13.
6. Cai J, Xu J, Lin D, et al. A Case Series of children with 2019 novel coronavirus infection: clinical and epidemiological features. Clin Infect Dis. 2020 Feb 28. pii: ciaa198. doi: 10.1093/cid/ciaa198. [Epub ahead of print]
7. Linton NM, Kobayashi T, Yang Y, et al. Incubation Period and Other Epidemiological Characteristics of 2019 Novel Coronavirus Infections with Right Truncation: A Statistical Analysis of Publicly Available Case Data. J Clin Med. 2020 Feb 17;9(2). pii: E538. doi: 10.3390/jcm9020538.
8. WHO News Release. Shortage of personal protective equipment endangering health workers worldwide. 2020 Mar 03.
9. Rey D. Post-exposure prophylaxis for HIV infection. Expert Rev Anti Infect Ther. 2011 Apr;9(4):431-442. doi: 10.1586/eri.11.20.
10. Chen J. Pathogenicity and transmissibility of 2019-nCoV-a quick overview and comparison with other emerging viruses. Microbes Infect. 2020. doi:10.1016/j.micinf.2020.01.004.
11. Hoffmann M, Kleine-Weber H, Schroeder S, et al. SARS-CoV-2 Cell Entry Depends on ACE2 and TMPRSS2 and Is Blocked by a Clinically Proven Protease Inhibitor. 2020 Mar 04; Cell. pii: S0092-8674(20)30229-4 (pre-publication). doi: 10.1016/j.cell.2020.02.052.
12. Li CC, Wang XJ, Wang HR. Repurposing host-based therapeutics to control coronavirus and influenza virus. Drug Discov Today. 2019 Mar;24(3):726-736. doi: 10.1016/j.drudis.2019.01.018. Epub 2019 Jan 31.
13. Lee JS, Adhikari NKJ, Kwon HY, et al. Anti-Ebola therapy for patients with Ebola virus disease: a systematic review. BMC Infect Dis. 2019 May 02;19(1):376. doi: 10.1186/s12879-019-3980-9.
14. Slater AF. Chloroquine: mechanism of drug action and resistance in *Plasmodium falciparum*. Pharmacol Ther. 1993 Feb-Mar;57(2-3):203-235.
15. Mackenzie AH. An appraisal of chloroquine. Arthritis Rheum. 1970 May-Jun;13(3):280-291.

16. Shippey EA, Wagler VD, Collamer AN. Hydroxychloroquine: an old drug with new relevance. Cleve Clin J Med. 2018 Jun;85(6):459-467. doi: 10.3949/ccjm.85a.17034
17. Schrezenmeier E, Dörner T. Mechanisms of action of hydroxychloroquine and chloroquine: implications for rheumatology. Nat Rev Rheumatol. 2020 Mar;16(3):155-166. doi: 10.1038/s41584-020-0372-x.
18. Rolain JM, Colson P, Raoult D. Recycling of chloroquine and its hydroxyl analogue to face bacterial, fungal and viral infections in the 21st century. Int J Antimicrob Agents. 2007 Oct;30(4):297-308.
19. Savarino A, Shytaj IL. Chloroquine and beyond: exploring anti-rheumatic drugs to reduce immune hyperactivation in HIV/AIDS. Retrovirology. 2015 Jun 18;12:51. doi: 10.1186/s12977-015-0178-0.
20. Keyaerts E, Vijgen L, Maes P, et al. *In vitro* inhibition of severe acute respiratory syndrome coronavirus by chloroquine. Biochem Biophys Res Commun. 2004 Oct;323(1):264-268.
21. Vincent MJ, Bergeron E, Benjannet S, et al. Chloroquine is a potent inhibitor of SARS coronavirus infection and spread. Virol J. 2005 Aug;2:69-78.
22. Wang M, Cao R, Zhang L, et al. Remdesivir and chloroquine effectively inhibit the recently emerged novel coronavirus (2019-nCoV) in vitro. Cell Res. 2020 Mar;30(3):269-271. doi: 10.1038/s41422-020-0282-0. Epub 2020 Feb 04.
23. Yao X, Ye F, Zhang M, et al. *In vitro* antiviral activity and projection of optimized dosing design of hydroxychloroquine for the treatment of severe acute respiratory syndrome coronavirus 2 (SARS-CoV-2). Clin Inf Dis. 2020 Mar 09. pii: ciaa237. doi: 10.1093/cid/ciaa237. [Epub ahead of print].
24. Gao J, Tian Z, Yang X. Breakthrough: chloroquine phosphate has shown apparent efficacy in treatment of COVID-19 associated pneumonia in clinical studies. Biosci Trends. 2020 Feb 19. doi: 10.5582/bst.2020.01047. [Epub ahead of print].
25. Furst DE, Lindsley H, Baethge B, et al. Dose-loading with hydroxychloroquine improves the rate of response in early, active rheumatoid arthritis: a randomized, double-blind six-week trial with eighteen-week extension. Arthritis Rheum. 1999 Feb;42(2):357-365.
26. Collins K, Jackson K, Gustafson D. Hydroxychloroquine: a physiologically based pharmacokinetic model in the context of cancer-related autophagy modulation. J Pharmacol Exp Ther. 2018;365(3):447-459.
27. Tett SE, Cutler DJ, Day RO, Brown KF. Bioavailability of hydroxychloroquine tablets in healthy volunteers. Br J Clin Pharmacol. 1989;27(6): 771-779.
28. Hu Z, Song C, Xu C, et al. Clinical characteristics of 24 asymptomatic infections with COVID-19 screened among close contacts in Nanjing, China. Sci China Life Sci. 2020 Mar 04. doi: 10.1007/s11427-020-1661-4. [Epub ahead of print].
29. Barrett B, Brown R, Rakel D, et al. Placebo effects and the common cold: a randomized controlled trial. Ann Fam Med. 2011 Jul-Aug;9(4):312-322. doi: 10.1370/afm.1250.
30. Fujii T, Luethi N, Young PJ, et al. Effect of vitamin C, hydrocortisone, and thiamine vs hydrocortisone alone on time alive and free of vasopressor support among patients with septic shock: the VITAMINS randomized clinical trial. JAMA. 2020 Jan 17. doi: 10.1001/jama.2019.22176. [Epub ahead of print].
31. Graat J, Schouten E, Kok F. Effect of daily vitamin E and multivitamin-mineral supplementation on acute respiratory tract infections in elderly persons: a randomized controlled trial. JAMA. 2002 Aug 14;288(6):715-721.

32. WHO. Report of the WHO-China joint mission on coronavirus disease 2019 (COVID-19). 2020 Feb 16-24.
33. Cao Y, Li L, Feng Z, Wan S, Huang P, Sun X, et al. Comparative genetic analysis of the novel coronavirus (2019-nCoV/SARS-CoV-2) receptor ACE2 in different populations. Cell Discovery. 2020; 6(1):11.
34. Bi Q, Yongsheng W, Shujiang M, et al. Epidemiology and transmission of COVID-19 in Shenzhen, China: analysis of 391 cases and 1286 of their close contacts. medRxiv. 2020. https://doi.org/10.1101/2020.03.03.20028423.
35. Srinivasa A, Tosounidou S, Gordon C. Increased Incidence of Gastrointestinal Side Effects in Patients Taking Hydroxychloroquine: A Brand-related Issue? J Rheumatol. 2017 Mar;44(3):398. doi: 10.3899/jrheum.161063.
36. UN3373 Medical Packaging. Biological Substance Category B. Available at: https://www.un3373.com/category-biological-substances/category-b. Updated 2018 Jun. Accessed 2020 Mar 01.

13. Appendices

Appendix 1: Abbreviations and Terms

Term	Definition
ACE2	Angiotensin converting enzyme 2
AE	Adverse event
COVID-19	Coronavirus disease
CQ	Chloroquine
DAIDS	Division of Acquired Immunodeficiency Syndrome
DAIDS	Division of acquired immunodeficiency syndrome
DBS	Dried blood spot
DSMB	Data and safety monitoring board
EC_{50}	Half-maximal effective concentration
eCRFs	Electronic case report forms
Eligible	Qualified for enrollment into the study based upon adherence to inclusion/exclusion criteria
GFR	Glomerular filtration rate
HCQ	Hydroxychloroquine
HIPAA	Health Insurance Portability and Accountability Act
IATA	International Air Transport Association
ICF	Informed consent form
Index case	Term used throughout the protocol to denote the person with confirmed or suspected SARS-CoV-2 infection to whom the study participant was exposed
IRB	Institutional Review Board
MERS-CoV	Middle East respiratory syndrome coronavirus
Participant(s)	Term used throughout the protocol to denote the enrolled individual(s)
PCR	Polymerase chain reaction
PEP	Post-exposure prophylaxis
PK	Pharmacokinetic
RNA	Ribonucleic acid
SAE	Serious adverse event
SARS-CoV	Severe acute respiratory syndrome coronavirus
SARS-CoV-2	Severe acute respiratory syndrome coronavirus 2
SoA	Schedule of Activities
WHO	World Health Organization
WIRB	Western Institutional Review Board

Appendix 2: Study Governance Considerations

Investigators and Institutional Affiliations

The following Investigators and Institutional Affiliations were established at the time of protocol authoring. Designees may be provided, as appropriate. Other institutions may utilize this model protocol with permission from a Co-Principal Investigator.

Name	Role	Institution	Contact Information
Dr. Ruanne V. Barnabas	Principal Investigator	University of Washington	Phone: +1 206 520 3813<br>Email: rbarnaba@uw.edu
Dr. Anna Bershteyn	Co-Principal Investigator	New York University	Phone: +1 408 309 9610<br>Email: Anna.Bershteyn@nyulangone.org
**University of Washington Coordinating Center**			
Dr. Elizabeth Brown	Study Statistician	Fred Hutchinson Cancer Research Center	Phone: +1 206 667-1731<br>Email: erbrown@fredhutch.org
Dr. Jared Baeten	Co-Investigator	University of Washington	Phone: +1 206 520-3808<br>Email: jbaeten@uw.edu
Dr. Connie Celum	Co-Investigator	University of Washington	Phone: +1 206 520-3800<br>Email: ccelum@uw.edu
Dr. Mark Wener	Co-Investigator	University of Washington	Phone: +1 206 598-6152<br>Email: wener@uw.edu
Dr. Anna Wald	Co-Investigator	University of Washington	Phone: +1 206 520-4340<br>Email: annawald@uw.edu
Dr. Helen Y. Chu	Co-Investigator	University of Washington	Phone: +1 206 685-5386<br>Email: helenchu@uw.edu

Collaborators from Bill & Melinda Gates Foundation

Name	Contact Information
Steve Kern, PhD	Phone: +1-206-770-2496<br>E-mail: steven.kern@gatesfoundation.org
Scott Miller, MD	Phone: +1-206-409-7757<br>E-mail: scott.miller@gatesfoundation.org
Peter Dull, MD	Phone: +1-206-770-2465<br>E-mail: peter.dull@gatesfoundation.org

Committees Structure

Study Team Monitoring

The study team will monitor the conduct of the study through monthly summary reports of arms of accrual and baseline characteristics and quarterly reports of data pooled over treatment arms of data completeness, specimen collection, and adverse events (AEs). The study will review individual participant-level safety data frequently to assess the relation of all reported AEs to study treatment. On a weekly basis, the study team will review by-arm summaries of premature study discontinuations and premature study treatment discontinuations (and reasons) and AEs.

Independent Monitor

Study conduct will be monitored by an independent monitor. Monitors will visit participating clinical research sites to review the individual participant records, including consent forms, electronic case report forms, supporting data, laboratory specimen records, and endpoints through laboratory and medical records (physicians' progress notes, nurses' notes, individuals' hospital charts), to ensure protection of study participants, compliance with the protocol, and accuracy and completeness of records. The monitors also will inspect sites' regulatory files to ensure that regulatory requirements are being followed and sites' pharmacies to review product storage and management.

Data and Safety Monitoring Board

An independent data and safety monitoring board (DSMB) will be convened for this study with expertise in coronavirus disease (COVID-19) or respiratory viruses, post-exposure prophylaxis (PEP), and emerging epidemics and a biostatistician. The purpose of the DSMB is to monitor the study for operational futility, social harms, and efficacy. The DSMB will evaluate the progress of the project, including periodic assessments of accrual, retention, safety, performance and variation of the project sites, and other factors that can affect project implementation.

The target number of households (index cases) and their contacts enrolled into the study will be re-assessed according to the distribution of cluster (household/institution) sizes seen in the study. The DSMB will review and approve modifications to the overall enrollment target. The DSMB will review the study after half to two-thirds of follow-up time with pre-specified stopping rules for efficacy and futility in terms of the efficacy of hydroxychloroquine (HCQ) PEP in reducing the incidence of SARS-CoV-2 infection overall and the ability of the study to meet its objectives. The DSMB will review severe acute respiratory syndrome coronavirus 2 (SARS-CoV-2) endpoints. The DSMB will consider factors external to the project when relevant information becomes available, such as policy changes or scientific developments that may have an impact on project implementation, safety, and integration of COVID-19 PEP into community-based disease prevention.

The DSMB will conduct interim reviews when adequate data have been accrued and convene by teleconference. Open reports containing accrual and retention rates, participant characteristics, and serious adverse events will be sent to the protocol team and DSMB members the week prior to the DSMB meeting. Only the DSMB members and the unblinded biostatistician will receive password-protected closed reports of SARS-CoV-2 endpoints by randomization arm.

Regulatory and Ethical Considerations

The study will be conducted according to Good Clinical Practice, the Belmont Report, and the Declaration of Helsinki. The study protocol, site-specific informed consent forms (ICFs), participant education and recruitment materials, and other requested documents—including any subsequent modifications—will be reviewed and approved by Western Institutional Review Board (WIRB), as the single IRB of record, responsible for oversight of research conducted at the study sites. Subsequent to initial review and approval, the WIRB will review the study at least annually.

Informed Consent Process

The principles of informed consent in the current edition of the Declaration of Helsinki will be implemented in each clinical study before any protocol-specified procedures or interventions are carried out. The consent form will describe the purpose of the study, the procedures to be followed, and the risks and benefits of participation. A copy of the consent form will be given to the participant, and this fact will be documented in the participant's record.

A participant who is rescreened is not required to sign another ICF; eligibility for the study must be re-checked prior to enrollment.

Study Records

Each study site will establish a standard operating procedure for confidentiality protection. Each site will ensure that study records including administrative documentation and regulatory documentation as well as documentation related to each participant enrolled in the study, including ICFs, locator forms, case report forms, notations of all contacts with the participant, and all other source documents are stored in a secure manner.

Confidentiality

Participants' study information will not be released without their written permission, except as necessary for oversight by:

- The protocol Co-Principal Investigators or designees
- Study funders
- WIRB
- New York University IRB
- University of Washington IRB

All laboratory specimens, evaluation forms, reports, and other records that leave the site will be identified by coded number only to maintain participant confidentiality. The exceptions are SARS-CoV-2 testing results which are subject to local and state reporting which is names-based. Local public health may contact participants diagnosed with SARS-CoV-2 for the purpose of surveillance and contact notification. Participants will be informed prior to SARS-CoV-2 testing that results are reportable and may lead to contact by local public health if results are positive for infection.

All records will be kept locked. All computer entry and networking programs will be done with coded numbers only. Clinical information will not be released without written permission of the participant, except as necessary for monitoring by the IRB, Office for Human Research

Protections, other local, United States, and international regulatory entities as part of their duties, or the industry supporters or designees.

Appendix 3: Site-specific Protocol Addendum Template

PURPOSE: The use of this site-specific protocol addendum is recommended with a multi-site study.

The purpose of this site-specific protocol addendum is to obtain information describing the local site-specific elements for conduct of the Core Protocol. The descriptions should focus on who, what, and where of the local study activities.

INSTRUCTIONS:

1. Add the Name of the study site to the Title of the Protocol in the header of the document.
2. Please complete each section below. If general description language already appears in the Core Protocol, which adequately describes the local activity at the study site, a notation can be inserted in the given section that reads, "As described in the Core Protocol."
3. Please identify the completed addendum document using a version number and date.
4. Please note: the site-specific protocol addendum will require review and approval by the local Institutional Review Board (IRB)/Ethics Committee (EC) of record for the study site.

Study Site Information:

Name of Institution/Company:

Address:

Study Site Information for the Site Investigator:

Name of Site Investigator:

Title:

Institution/Affiliation:

Address:

Telephone Number:

Cell/Other Number:

Fax Number:

Study Site Number:

Assurance Information:

United States Department of Health and Human Services Office for Human Research Protections Assurance Federal Wide Assurance number:

Expiration Date:

IRB/Ethics Committee Information:

Name of Human Subjects Protection oversight office for study site:

Name and number of reviewing IRB/EC:

Telephone Number at Office:

Fax Number at Office:

If Available, Point of Contact at IRB/EC:

Name:

Telephone Number:

Local Site-Specific Information:

1. Identify key study personnel (include name, title, address, point of contact information).
2. Description of key study personnel roles and responsibilities.
3. Describe the local recruiting procedures and strategies. Provide copy of any site-specific recruitment material. Identification of personnel responsible for completing tasks.
4. Describe the local consenting process. Provide a copy of the site-specific consent form(s). If an Ombudsman is named for the study site, provide name, title, and point of contact information.
5. Identify local study collaborations at the site such as pharmacy, laboratories, and other institutional departments.
6. Describe the local specimen/sampling procedures in place. Include acquisition, disposition, storage, and unique coding. If samples will be kept for future use, describe procedures and the security measures for short-term and long-term management. Provide name of repository.
7. Describe the plan for on-site management of study records and data, and participant study records. Explain procedures and security measures in place for short-term and long-term management. Declare who will have access to data.
8. Describe the local measures in place to promote privacy and confidentiality.
9. Describe the local procedures in place for provision of care for the participant regarding research-related injuries.
10. Identify who the participant can contact locally should the participant have any questions regarding the research. Identify who the participant can contact should the participant have questions regarding their rights as a study participant. Include the points of contact information (name, title, and telephone number).
11. Describe the procedures in place to address Health Insurance Portability and Accountability Act requirements. If a separate authorization form will be used at the

study site, provide a copy or ensure appropriate language has been included in the consent document(s).

12. Describe any unique site-specific study procedures or supplemental activities.
13. Declare any unique study population/cultural influences, socioeconomic conditions, etc.
14. Declare any other site-specific reporting obligations and procedures. Name any additional oversight boards or committees.
15. Define abbreviations that may apply to the specific study site.

Appendix 4: Hydroxychloroquine Label

Generic hydroxychloroquine label (current as of June 2018) is available online at https://dailymed.nlm.nih.gov/dailymed/drugInfo.cfm?setid=b82bbda6-64f2-4426-b4ec-254eeea895ae and provided below.

HYDROXYCHLOROQUINE SULFATE- hydroxychloroquine sulfate tablet, film coated
Sandoz Inc

Hydroxychloroquine Sulfate Tablets, USP
Rx Only

DESCRIPTION

Hydroxychloroquine sulfate, USP is a colorless crystalline solid, soluble in water to at least 20 percent; chemically the drug is 2-[[4-[(7-Chloro-4-quinolyl) amino] pentyl] ethylamino] ethanol sulfate (1:1). Hydroxychloroquine sulfate has the following structural formula:

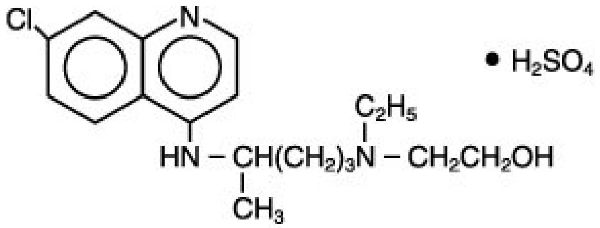

$C_{18}H_{26}ClN_3O \bullet H_2SO_4$ M.W. 433.96

Each tablet for oral administration contains 200 mg hydroxychloroquine sulfate, USP (equivalent to 155 mg hydroxychloroquine). Inactive ingredients: Dibasic calcium phosphate, hydroxypropyl cellulose, hydroxypropyl methylcellulose, magnesium stearate, polyethylene glycol, povidone, sodium bicarbonate, and titanium dioxide.

CLINICAL PHARMACOLOGY

Pharmacokinetics

Following a single 200 mg oral dose of hydroxychloroquine sulfate to healthy males, the mean peak blood concentration of hydroxychloroquine was 129.6 ng/mL, reached in 3.26 hours with a half-life of 537 hours (22.4 days). In the same study, the plasma peak concentration was 50.3 ng/mL reached in 3.74 hours with a half-life of 2963 hours (123.5 days). Urine hydroxychloroquine levels were still detectable after 3 months with approximately 10% of the dose excreted as the parent drug. Results following a single dose of a 200 mg tablet versus i.v. infusion (155 mg), demonstrated a half-life of about 40 days and a large volume of distribution. Peak blood concentrations of metabolites were observed at the same time as peak levels of hydroxychloroquine. The mean fraction of the dose absorbed was 0.74. After administration of single 155 mg and 310 mg intravenous doses, peak blood concentrations ranged from 1161 ng/mL to 2436 ng/mL (mean 1918 ng/mL) following the 155 mg infusion and 6 months following the 310 mg infusion. Pharmacokinetic parameters were not significantly different over the therapeutic dose range of 155 mg and 310 mg indicating linear kinetics.

Following chronic oral administration of hydroxychloroquine, significant levels of three metabolites, desethylhydroxychloroquine (DHCQ), desethylchloroquine (DCQ), and bidesethylhydroxychloroquine (BDCQ) have been found in plasma and blood, with DHCQ being the major metabolite. The absorption half-life was approximately 3 to 4 hours and the terminal half-life ranged from 40 to 50 days. The long half-life can be attributed to extensive tissue uptake rather than through decreased excretion. Peak plasma levels of hydroxychloroquine were seen in about 3 to 4 hours. Renal clearance in rheumatoid arthritis (RA) patients taking hydroxychloroquine sulfate for at least six months seemed to be similar to that of the single dose studies in volunteers, suggesting that no change occurs with chronic dosing. Range for renal clearance of unchanged drug was approximately 16 to 30% and did not correlate with

creatinine clearance; therefore, a dosage adjustment is not required for patients with renal impairment. In RA patients, there was large variability as to the fraction of the dose absorbed (i.e. 30 to 100%), and mean hydroxychloroquine levels were significantly higher in patients with less disease activity. Cellular levels of patients on daily hydroxychloroquine have been shown to be higher in mononuclear cells than polymorphonuclear leucocytes.

Microbiology – Malaria

Mechanism of Action

The precise mechanism by which hydroxychloroquine exhibits activity against *Plasmodium* is not known. Hydroxychloroquine, like chloroquine, is a weak base and may exert its effect by concentrating in the acid vesicles of the parasite and by inhibiting polymerization of heme. It can also inhibit certain enzymes by its interaction with DNA.

Activity In Vitro and in Clinical Infections

Hydroxychloroquine is active against the erythrocytic forms of chloroquine sensitive strains of *Plasmodium falciparum*, *Plasmodium malariae*, *Plasmodium ovale*, and *Plasmodium vivax*. Hydroxychloroquine is not active against the gametocytes and exoerythrocytic forms including the hypnozoite stage (*P. vivax* and *P. ovale*) of the *Plasmodium* parasites.

Drug Resistance

P. falciparum strains exhibiting reduced susceptibility to chloroquine also show reduced susceptibility to hydroxychloroquine.

Resistance of *Plasmodium* parasites to chloroquine is widespread (see INDICATIONS AND

USAGE – Malaria).

Patients in whom chloroquine or hydroxychloroquine have failed to prevent or cure clinical malaria or parasitemia, or patients who acquired malaria in a geographic area where chloroquine resistance is known to occur should be treated with another form of antimalarial therapy (see INDICATIONS AND USAGE – Malaria and WARNINGS).

Rheumatoid Arthritis and Systemic Lupus Erythematosus

Mechanism of Action

The mechanisms underlying the anti-inflammatory and immunomodulatory effects of hydroxychloroquine sulfate are unknown.

INDICATIONS AND USAGE

Malaria

Hydroxychloroquine sulfate is indicated for the treatment of uncomplicated malaria due to *P. falciparum, P. malariae, P. ovale,* and *P. vivax.*

Hydroxychloroquine sulfate is indicated for the prophylaxis of malaria in geographic areas where chloroquine resistance is not reported.

Limitations of Use in Malaria

• Hydroxychloroquine sulfate is not recommended for the treatment of complicated malaria.

• Hydroxychloroquine sulfate is not effective against chloroquine or hydroxychloroquine-resistant strains of *Plasmodium* species (see CLINICAL PHARMACOLOGY – Microbiology). Hydroxychloroquine sulfate is not recommended for the treatment of malaria acquired in geographic areas where chloroquine resistance occurs or when the *Plasmodium* species has not been identified.

• Hydroxychloroquine sulfate is not recommended for malaria prophylaxis in geographic areas where

chloroquine resistance occurs.

• Hydroxychloroquine sulfate does not prevent relapses of *P. vivax* or *P. ovale* because it is not active against the hypnozoite forms of these parasites. For radical cure of *P. vivax* and *P. ovale* infections, concomitant therapy with an 8-aminoquinoline compound is necessary (see CLINICAL PHARMACOLOGY – Microbiology).

Prior to prescribing hydroxychloroquine sulfate for the treatment or prophylaxis of malaria, consult the Centers for Disease Control and Prevention (CDC) Malaria website (http://www.cdc.gov/malaria).

Lupus Erythematosus

Hydroxychloroquine sulfate is indicated for the treatment of chronic discoid lupus erythematosus and systemic lupus erythematosus in adults.

Rheumatoid Arthritis

Hydroxychloroquine sulfate is indicated for the treatment of acute and chronic rheumatoid arthritis in adults.

CONTRAINDICATIONS

Use of hydroxychloroquine sulfate is contraindicated in patients with known hypersensitivity to 4-aminoquinoline compounds.

WARNINGS

Resistant Strains of Malaria

Hydroxychloroquine sulfate is not effective against chloroquine-resistant strains of *P. falciparum* (see CLINICAL PHARMACOLOGY – Microbiology).

Ocular

Irreversible retinal damage has been observed in some patients who had received hydroxychloroquine sulfate. Significant risk factors for retinal damage include daily doses of hydroxychloroquine sulfate >6.5 mg/kg (5 mg/kg base) of actual body weight, durations of use >5 years, subnormal glomerular filtration, use of some concomitant drug products such as tamoxifen citrate and concurrent macular disease.

A baseline ocular examination is recommended within the first year of starting hydroxychloroquine sulfate. The baseline exam should include: best corrected distance visual acuity (BCVA), an automated threshold visual field (VF) of the central 10 degrees (with retesting if an abnormality is noted), and spectral domain ocular coherence tomography (SD-OCT).

For individuals with significant risk factors (daily dose of hydroxychloroquine sulfate >5 mg/kg base of actual body weight, subnormal glomerular filtration, use of tamoxifen citrate or concurrent macular disease) monitoring should include annual examinations which include BCVA, VF and SD-OCT. For individuals without significant risk factors, annual exams can usually be deferred until five years of treatment.

In individuals of Asian descent, retinal toxicity may first be noticed outside the macula. In patients of Asian descent, it is recommended that visual field testing be performed in the central 24 degrees instead of the central 10 degrees.

It is recommended that hydroxychloroquine be discontinued if ocular toxicity is suspected and the patient should be closely observed given that retinal changes (and visual disturbances) may progress even after cessation of therapy.

Cardiac Effects, Including Cardiomyopathy and QT Prolongation

Postmarketing cases of life-threatening and fatal cardiomyopathy have been reported with use of hydroxychloroquine sulfate as well as with use of chloroquine. Patients may present with atrioventricular block, pulmonary hypertension, sick sinus syndrome or with cardiac complications. ECG findings may include atrioventricular, right or left bundle branch block. Signs or symptoms of cardiac compromise have appeared during acute and chronic treatment. Clinical monitoring for signs and symptoms of cardiomyopathy is advised, including use of appropriate diagnostic tools such as ECG to monitor patients for cardiomyopathy during hydroxychloroquine sulfate therapy. Chronic toxicity should be considered when conduction disorders (bundle branch block/atrio-ventricular heart block) or biventricular hypertrophy are diagnosed. If cardiotoxicity is suspected, prompt discontinuation of hydroxychloroquine sulfate may prevent life-threatening complications.

Hydroxychloroquine sulfate prolongs the QT interval. Ventricular arrhythmias and *torsades de pointes* have been reported in patients taking hydroxychloroquine sulfate (see OVERDOSAGE). Therefore, hydroxychloroquine sulfate should not be administered with other drugs that have the potential to prolong the QT interval (see DRUG INTERACTIONS).

Worsening of Psoriasis and Porphyria

Use of hydroxychloroquine sulfate in patients with psoriasis may precipitate a severe attack of psoriasis. When used in patients with porphyria the condition may be exacerbated. The preparation should not be used in these conditions unless in the judgment of the physician the benefit to the patient outweighs the possible hazard.

Proximal Myopathy and Neuropathy

Skeletal muscle myopathy or neuropathy leading to progressive weakness and atrophy of proximal muscle groups, depressed tendon reflexes, and abnormal nerve conduction, have been reported. Muscle and nerve biopsies have been associated with curvilinear bodies and muscle fiber atrophy with vacuolar changes. Assess muscle strength and deep tendon reflexes periodically in patients on long-term therapy with hydroxychloroquine sulfate.

Neuropsychiatric Events, Including Suicidality

Suicidal behavior has been rarely reported in patients treated with hydroxychloroquine sulfate.

Hypoglycemia

Hydroxychloroquine sulfate has been shown to cause severe hypoglycemia including loss of consciousness that could be life threatening in patients treated with or without antidiabetic medications (see DRUG INTERACTIONS and ADVERSE REACTIONS). Patients treated with hydroxychloroquine sulfate should be warned about the risk of hypoglycemia and the associated clinical signs and symptoms. Patients presenting with clinical symptoms suggestive of hypoglycemia during treatment with hydroxychloroquine sulfate should have their blood glucose checked and treatment reviewed as necessary.

PRECAUTIONS

General

Use with caution in patients with gastrointestinal, neurological, or blood disorders, and in those with a sensitivity to quinine.

Hepatic/Renal Disease

Antimalarial compounds should be used with caution in patients with hepatic disease or alcoholism or in conjunction with known hepatotoxic drugs. A reduction in dosage may be necessary in patients with hepatic or renal disease, as well as in those taking medicines known to affect these organs.

Hematologic Effects/Laboratory Tests

Antimalarial compounds should be used with caution in patients with hepatic disease or alcoholism or in conjunction with known hepatotoxic drugs. Periodic blood cell counts should be performed if patients are given prolonged therapy. If any severe blood disorder such as aplastic anemia, agranulocytosis, leukopenia, or thrombocytopenia, appears which is not attributable to the disease under treatment, consider discontinuation of hydroxychloroquine sulfate.

Hydroxychloroquine sulfate should be administered with caution in patients having glucose-6-phosphate dehydrogenase (G-6-PD) deficiency.

Dermatologic Effects

Dermatologic reactions to hydroxychloroquine sulfate may occur and, therefore, proper care should be exercised when it is administered to any patient receiving a drug with a significant tendency to produce dermatitis.

Drug Interactions

Digoxin

Concomitant hydroxychloroquine sulfate and digoxin therapy may result in increased serum digoxin levels: serum digoxin levels should be closely monitored in patients receiving combined therapy.

Insulin or Antidiabetic Drugs

As hydroxychloroquine sulfate may enhance the effects of a hypoglycemic treatment, a decrease in doses of insulin or antidiabetic drugs may be required.

Drugs That Prolong QT Interval and Other Arrhythmogenic Drugs

Hydroxychloroquine sulfate prolongs the QT interval and should not be administered with other drugs that have the potential to induce cardiac arrhythmias. Also, there may be an increased risk of inducing ventricular arrhythmias if hydroxychloroquine sulfate is used concomitantly with other arrhythmogenic drugs.

Mefloquine and Other Drugs Known to Lower the Convulsive Threshold

Hydroxychloroquine sulfate can lower the convulsive threshold. Co-administration of hydroxychloroquine sulfate with other antimalarials known to lower the convulsion threshold (e.g., mefloquine) may increase the risk of convulsions.

Antiepileptics

The activity of antiepileptic drugs might be impaired if co-administered with hydroxychloroquine sulfate.

Methotrexate

Combined use of methotrexate with hydroxychloroquine sulfate has not been studied and may increase the incidence of adverse effects.

Cyclosporin

An increased plasma cyclosporin level was reported when cyclosporin and hydroxychloroquine sulfate were co-administered.

The following interactions have been observed on treatment with the structurally related substancechloroquine phosphate, and therefore cannot be ruled out for hydroxychloroquine.

Praziquantel

Chloroquine has been reported to reduce the bioavailability of praziquantel.

Antacids and Kaolin

Antacids and kaolin can reduce absorption of chloroquine; an interval of at least 4 hours between intake of these agents and chloroquine should be observed.

Cimetidine

Cimetidine can inhibit the metabolism of chloroquine, increasing its plasma level.

Concomitant use of cimetidine should be avoided.

Ampicillin

In a study of healthy volunteers, chloroquine significantly reduced the bioavailability of ampicillin.

Information for Patients

Patients should be informed of the early signs and symptoms of toxicity such as rash or visual changes. Patients must see their physicians promptly in case of the appearance of these or of any unusual effects. Periodic laboratory tests may be recommended in some patients. Patients should be fully informed of the potential risks of the use of hydroxychloroquine sulfate, especially in pregnancy and in children.

Carcinogenesis, Mutagenesis, Impairment of Fertility

Long-term studies in animals have not been conducted to evaluate the carcinogenic potential of hydroxychloroquine sulfate.

The mutagenic potential of hydroxychloroquine was not evaluated. However, chloroquine has been shown to be a catalytic inhibitor of DNA repair enzymes (topoisomerase II) and to produce weak genotoxic effects through this mode of action.

Pregnancy

Teratogenic Effects

Human pregnancies resulting in live births have been reported in the literature and no increase in the rate of birth defects has been demonstrated. Embryonic deaths and malformations of anophthalmia and microphthalmia in the offspring have been reported when pregnant rats received large doses of chloroquine.

Nursing Mothers

Caution should be exercised when administering hydroxychloroquine sulfate to nursing women. It has been demonstrated that hydroxychloroquine administered to nursing women is excreted in human milk and it is known that infants are extremely sensitive to the toxic effects of 4-aminoquinolines.

Pediatric Use

Safety and efficacy have not been established in the chronic use of hydroxychloroquine sulfate for systemic lupus erythematosus and juvenile idiopathic arthritis in children. Children are especially sensitive to the 4-aminoquinoline compounds. Most reported fatalities followed the accidental ingestion of chloroquine, sometimes in small doses (0.75 g or 1 g in one 3-year-old child). Patients should be strongly warned to keep these drugs out of the reach of children (see OVERDOSAGE).

Geriatric Use

Clinical studies of hydroxychloroquine sulfate did not include sufficient numbers of subjects aged 65 and over to determine whether they respond differently from younger subjects. However, this drug is known to be substantially excreted by the kidney, and the risk of toxic reactions to this drug may be greater in patients with impaired renal function. Because elderly patients are more likely to have decreased renal function, care should be taken in dose selection and it may be useful to monitor renal function.

ADVERSE REACTIONS

The following adverse reactions have been identified during post-approval use of hydroxychloroquine sulfate or other 4-aminoqunoline compounds. Because these reactions are reported voluntarily from a

population of uncertain size, it is not always possible to reliably estimate their frequency or establish a causal relationship to drug exposure.

Blood and Lymphatic System Disorders

Bone marrow failure, anemia, aplastic anemia, agranulocytosis, leukopenia, and thrombocytopenia. Hemolysis reported in individuals with glucose-6-phosphate dehydrogenase (G-6-PD) deficiency.

Cardiac Disorders

Cardiomyopathy which may result in cardiac failure and in some cases a fatal outcome (see WARNINGS and OVERDOSAGE). Hydroxychloroquine sulfate prolongs the QT interval. Ventricular arrhythmias and *torsades de pointes* have been reported in patients taking hydroxychloroquine sulfate (see OVERDOSAGE and DRUG INTERACTIONS).

Ear and Labyrinth Disorders

Vertigo, tinnitus, nystagmus, nerve deafness, deafness.

Eye Disorders

Irreversible retinopathy with retinal pigmentation changes (bull’s eye appearance), visual field defects (paracentral scotomas) and visual disturbances (visual acuity), maculopathies (macular degeneration), decreased dark adaptation, color vision abnormalities, corneal changes (edema and opacities) including corneal deposition of drug with or without accompanying symptoms (halo around lights, photophobia, blurred vision).

Gastrointestinal Disorders

Nausea, vomiting, diarrhea, and abdominal pain.

General Disorders and Administration Site Conditions

Fatigue.

Hepatobiliary Disorders

Liver function tests abnormal, hepatic failure acute.

Immune System Disorders

Urticaria, angioedema, bronchospasm.

Metabolism and Nutrition Disorders

Decreased appetite, hypoglycemia, porphyria, weight decreased.

Musculoskeletal and Connective Tissue Disorders

Sensorimotor disorder, skeletal muscle myopathy or neuromyopathy leading to progressive weakness and atrophy of proximal muscle groups, depression of tendon reflexes and abnormal nerve conduction.

Nervous System Disorders

Headache, dizziness, seizure, ataxia and extrapyramidal disorders such as dystonia, dyskinesia, and tremor have been reported with this class of drugs.

Psychiatric Disorders

Affect/emotional lability, nervousness, irritability, nightmares, psychosis, suicidal behavior.

Skin and Subcutaneous Tissue Disorders

Rash, pruritus, pigmentation disorders in skin and mucous membranes, hair color changes, alopecia. Dermatitis bullous eruptions including erythema multiforme, Stevens-Johnson syndrome, and toxic epidermal necrolysis, drug reaction with eosinophilia and systemic symptoms (DRESS syndrome), photosensitivity, dermatitis exfoliative, acute generalized exanthematous pustulosis (AGEP). AGEP has

to be distinguished from psoriasis, although hydroxychloroquine sulfate may precipitate attacks of psoriasis. It may be associated with pyrexia and hyperleukocytosis.

To report SUSPECTED ADVERSE REACTIONS, contact Sandoz Inc. at 1-800-525-8747 or FDA at 1-800-FDA-1088 or www.fda.gov/medwatch.

OVERDOSAGE

The 4-aminoquinoline compounds are very rapidly and completely absorbed after ingestion, and in accidental overdosage, or rarely with lower doses in hypersensitive patients, toxic symptoms may occur within 30 minutes. The symptoms of overdosage may include headache, drowsiness, visual disturbances, cardiovascular collapse, convulsions, hypokalemia, rhythm and conduction disorders including QT prolongation, *torsades de pointes*, ventricular tachycardia and ventricular fibrillation, followed by sudden potentially fatal respiratory and cardiac arrest. Treatment is symptomatic and must be prompt. Immediate gastric lavage until the stomach is completely emptied is indicated. After lavage, activated charcoal is introduced by the stomach tube within 30 minutes of ingestion of the drug may inhibit further intestinal absorption. To be effective, the dose of activated charcoal should be at least five times the estimated dose of hydroxychloroquine ingested.

Consideration should be given to administering diazepam parenterally since studies suggest that it may be beneficial in reversing chloroquine and hydroxychloroquine cardiotoxicity.

Respiratory support and shock management should be instituted as necessary.

Exchange transfusions are used to reduce the level of 4-aminoquinoline drug in the blood.

A patient who survives the acute phase and is asymptomatic should be closely observed for at least six hours. Fluids may be forced and sufficient ammonium chloride (8 g daily in divided doses for adults) may be administered for a few days to acidify the urine. This will promote urinary excretion in cases of both overdosage and sensitivity. However, caution must be exercised in patients with impaired renal function and/or metabolic acidosis.

DOSAGE AND ADMINISTRATION

One tablet of 200 mg of hydroxychloroquine sulfate is equivalent to 155 mg base. Take hydroxychloroquine sulfate with a meal or a glass of milk.

Malaria

Prophylaxis

Adults

400 mg (310 mg base) once weekly on the same day of each week starting 2 weeks prior to exposure, and continued for 4 weeks after leaving the endemic area.

Weight-Based Dosing in Adults and Pediatric Patients

6.5 mg/kg (5 mg/kg base), not to exceed 400 mg (310 mg base), once weekly on the same day of the week starting 2 weeks prior to exposure, and continued for 4 weeks after leaving the endemic area.

Treatment of Uncomplicated Malaria

Adults

800 mg (620 mg base) followed by 400 mg (310 mg base) at 6 hours, 24 hours and 48 hours after the initial dose (total 2000 mg hydroxychloroquine sulfate or 1550 mg base).

Weight-Based Dosage in Adults and Pediatric Patients

13 mg/kg (10 mg/kg base), not to exceed 800 mg (620 mg base) followed by 6.5 mg/kg (5 mg/kg base), not to exceed 400 mg (310 mg base), at 6 hours, 24 hours and 48 hours after the initial dose.

Hydroxychloroquine sulfate film-coated tablets cannot be divided, therefore they should not be used to treat patients who weigh less than 31 kg.

For radical cure of *P. vivax* and *P. malariae* infections, concomitant therapy with an 8-aminoquinoline compound is necessary.

Lupus Erythematosus

The recommended adult dosage is 200 to 400 mg (155 to 310 mg base) daily, administered as a single daily dose or in two divided doses. Doses above 400 mg a day are not recommended.

The incidence of retinopathy has been reported to be higher when this maintenance dose is exceeded.

Rheumatoid Arthritis

The action of hydroxychloroquine is cumulative and may require weeks to months to achieve the maximum therapeutic effect (see CLINICAL PHARMACOLOGY**).**

Initial Adult Dosage

400 mg to 600 mg (310 to 465 mg base) daily, administered as a single daily dose or in two divided doses. In a small percentage of patients, side effects may require temporary reduction of the initial dosage.

Maintenance Adult Dosage

When a good response is obtained, the dosage may be reduced by 50 percent and continued at a maintenance level of 200 mg to 400 mg (155 to 310 mg base) daily, administered as a single daily dose or in two divided doses.

Do not exceed 600 mg or 6.5 mg/kg (5 mg/kg base) per day, whichever is lower, as the incidence of retinopathy has been reported to be higher when this maintenance dose is exceeded.

Corticosteroids and salicylates may be used in conjunction with hydroxychloroquine sulfate, and they can generally be decreased gradually in dosage or eliminated after a maintenance dose of hydroxychloroquine sulfate has been achieved.

HOW SUPPLIED

Hydroxychloroquine sulfate tablets, USP for oral administration are available as:

200 mg: Round, Off White, Standard Cup Tablet Core, Debossed GG 260 on One Side and Plain on the Reverse Side, Film Coated White

NDC 0781-5994-01 bottles of 100

NDC 0781-5994-05 bottles of 500

Each tablet contains 200 mg hydroxychloroquine sulfate, USP (equivalent to 155 mg base).

Store at 20° to 25°C (68° to 77°F) [see USP Controlled Room Temperature].

Dispense in a tight, light-resistant container.

KEEP OUT OF THE REACH OF CHILDREN.

Manufactured by

Sandoz Inc.

Princeton, NJ 08540

Rev. March 2018

MF5994REV03/18

200 mg Label

NDC 0781-5994-01

Hydroxychloroquine

Sulfate Tablets, USP

200 mg

Rx only

100 Tablets

SANDOZ

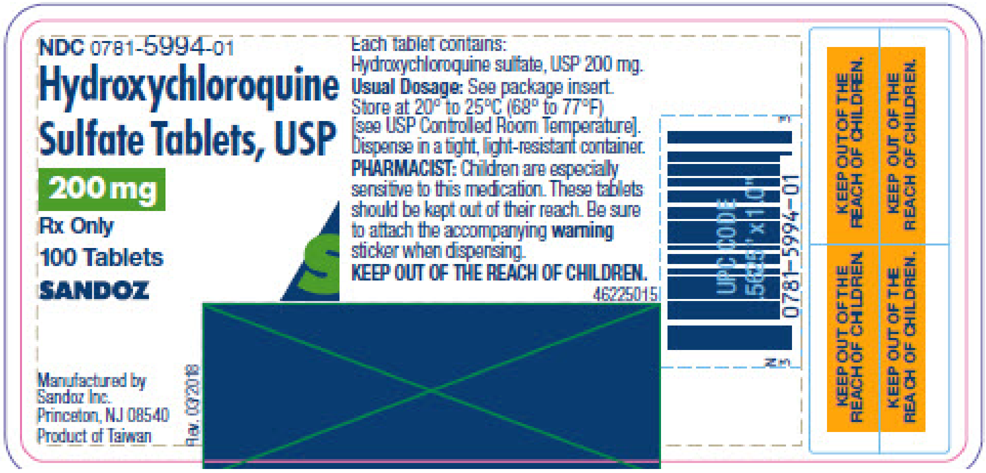

HYDROXYCHLOROQUINE SULFATE

hydroxychloroquine sulfate tablet, film coated

Product Information			
**Product Type**	HUMAN PRESCRIPTION DRUG	**Item Code (Source)**	NDC:0781-5994
**Route of Administration**	ORAL		

Active Ingredient/Active Moiety		
**Ingredient Name**	**Basis of Strength**	**Strength**
**HYDROXYCHLOROQUINE SULFATE** (UNII: 8Q2869CNVH) (HYDROXYCHLOROQUINE - UNII:4QWG6N8QKH)	HYDROXYCHLOROQUINE SULFATE	200 mg

Inactive Ingredients	
**Ingredient Name**	**Strength**
**HYDROXYPROPYL CELLULOSE, UNSPECIFIED** (UNII: 9XZ8H6N6OH)	
**HYPROMELLOSE, UNSPECIFIED** (UNII: 3NXW29V3WO)	
**MAGNESIUM STEARATE** (UNII: 70097M6I30)	
**POLYETHYLENE GLYCOL, UNSPECIFIED** (UNII: 3WJQ0SDW1A)	
**POVIDONE, UNSPECIFIED** (UNII: FZ989GH94E)	
**SODIUM BICARBONATE** (UNII: 8MDF5V39QO)	

**TITANIUM DIOXIDE** (UNII: 15FIX9V2JP)	
**ANHYDROUS DIBASIC CALCIUM PHOSPHATE** (UNII: L11K75P92J)	

Product Characteristics			
**Color**	WHITE (Off-White)	**Score**	no score
**Shape**	ROUND	**Size**	9mm
**Flavor**		**Imprint Code**	GG;260
**Contains**			

Packaging

#	Item Code	Package Description	Marketing Start Date	Marketing End Date
**1**	NDC:0781-5994-01	100 in 1 BOTTLE; Type 0: Not a Combination Product	03/07/2018	
**2**	NDC:0781-5994-05	500 in 1 BOTTLE; Type 0: Not a Combination Product	03/07/2018	

Marketing Information

Marketing Category	Application Number or Monograph Citation	Marketing Start Date	Marketing End Date
ANDA	ANDA040104	11/30/1995	

Labeler - Sandoz Inc (005387188)

Revised: 3/2018 Sandoz Inc

Appendix 5: Physiologically Based Pharmacokinetic Modeling of Hydroxychloroquine Used for Post-exposure Prophylaxis

Peking University Third Hospital Drug Clinical Trial Center

Clinical Pharmacology and Pharmacometrics Office

Hydroxychloroquine Physiologically-based pharmacokinetic model (PBPK) Analysis Report

**Research Name**	**Hydroxychloroquine physiologically-based pharmacokinetic model (PBPK) analysis report (refer as PBPK report)**
**Report Number**	**BYSY-DCTC-CPPO-CQ&HCQ-PBPKAR-02**
**Drug**	**Hydroxychloroquine**
**Requested by**	**Bill & Melinda Gates Foundation**
**Version Number**	**1.0**
**PBPK Research Unit**	**Clinical Pharmacology and Pharmacometrics Office, Drug Clinical Trial Center, Peking University Third Hospital**
**PI**	**Dr. Dongyang Liu**
**Project Leader**	**Xueting Yao**
**E-mail**	**liangmuxueting@sina.com**
**Address**	**No. 49, Huayuan North Road, Haidian District, Beijing, China**
**Postal Code**	**100191**
**Report Date**	**20/03/2020**

Research Location and Researchers

Research Unit: Clinical Pharmacology and Pharmacometrics Office，Drug Clinical Trial Center , Peking University Third Hospital

Address: No. 49, Huayuan North Road, Haidian District, Beijing, China

Postal code: 100191

Telephone: +86-010-82265509

Researcher	Name	Telephone
Principal Investigator	Dongyang Liu<br>PhD，Associate Professor	+86-010-82265509
Project Leader	Xueting Yao<br>PhD	+86-010-82265509
PBPK Analyst	Zhe Hou<br>Master Degree Candidate	+86-010-82265509
Quality Control	Zihan Lei<br>Research Assistant	+86-010-82265509

Pharmacometrics Research Compliance Statement

This pharmacometrics research was complied with the Good Clinical Practice of the National Medical Products Administration (NMPA), the Standard Operation Procedure (SOP) of Clinical Pharmacology and Pharmacometrics Office, Drug Clinical Trial Center, Peking University Third Hospital, and rules of the NMPA and regional regulators.

Xueting Yao
2020.03.20 14:06:07 +08'00'

Xueting Yao, Project Leader Date

PhD

Clinical Pharmacology and Pharmacometrics Office

Drug Clinical Trial Center, Peking University Third Hospital

Zhe Hou
2020.03.20 14:28:57 +08'00'

Zhe Hou, PBPK Analyst Date

Master Degree candidate

Clinical Pharmacology and Pharmacometrics Office

Drug Clinical Trial Center, Peking University Third Hospital

Quality Assurance Statement

This pharmacometrics research report has been audited and inspected, and all the quality control results have been reported to the leader of pharmacometrics research and pharmacometrics researchers. This report accurately described the raw data from the research. All the original data and supporting files in this research were available and archived properly in the Drug Clinical Trial Center, Peking University Third Hospital.

Zihan Lei
2020.03.20 14:35:01 +08'00'

Zihan Lei Quality control Date

Research Assistant

Clinical Pharmacology and Pharmacometrics Office

Drug Clinical Trial Center, Peking University Third Hospital

Approval of Pharmacometrics Research Report

We reviewed and approved this pharmacometrics research report.

刘东阳

数字签名者：刘东阳
DN：cn=刘东阳, o=北医三院药物临床试验机构, ou=临床药理与定量药理学研究室, email=liudongyang@vip.sina.com, c=CN
日期：2020.03.20 14:41:27 +08'00'

Dongyang Liu, Principal Investigator — Date

PhD, Associate Professor

Director of Clinical Pharmacology and Pharmacometrics Office

Drug Clinical Trial Center, Peking University Third Hospital

Contents

Research Location and Researchers 2
Pharmacometrics Research Compliance Statement 3
Quality Assurance Statement 4
Approval of Pharmacometrics Research Report 5
1.Objectives 7
2.Introduction 7
3.Methods 7
    3.1 Data source 7
        3.1.1 Data for compound files 7
        3.1.2 Data used in model verification 7
    3.2 PBPK model research methods 8
        3.2.1 PBPK model research procedures 8
        3.2.2 Softwares and hardwares 8
        3.2.3 PBPK model development 8
        3.2.4 PBPK model verification 8
4.Results and conclusions 9
    4.1 PBPK model development and verification 9
    4.2 Simulation results 9
5.Appendix 14

1. Objectives

To simulate hydroxychloroquine (HCQ) concentration-time profiles in plasma, whole blood, and lung in Chinese healthy populations, Caucasian healthy populations and Caucasian renal injury with GFR 30-60 mL/min to support the study design of prophylactic use of HCQ.

2. Introduction

Since the novel coronavirus COVID-19 first emerged in December 2019, the epidemic has grown rapidly and globally. There have been many confirmed infections in numerous countries. Chloroquine phosphate and hydroxychloroquine sulfate have been shown to effectively suppress SARS-CoV-2 *in vitro* assay. This study aims to develop a PBPK modeling to understand the drug exposure in various tissues under different treatment regimens of chloroquine phosphate and hydroxychloroquine sulfate, and use the PBPK model to predict the drug concentration in site of action, namely lung tissue. The model is useful for optimizing dosage and dosing regimen selection for patients infected with SARS-CoV-2, and will support the clinical use of chloroquine phosphate and hydroxychloroquine sulfate in treatment of COVID-2019.

This report summarizes PBPK simulations under different dosing regimens of HCQ in Chinese healthy populations, Caucasian healthy populations and Caucasian renal injury with GFR 30-60 mL/min.

3. Methods

3.1 Data source

3.1.1 Data for compound files

The hydroxychloroquine parameters were obtained from published articles.

3.1.2 Data used in model verification

The published human pharmacokinetics data of hydroxychloroquine were collected from literatures.

A total of 16 articles related to human pharmacokinetics of hydroxychloroquine were collected according to the retrieval method, and were included.

3.2 PBPK model research methods

3.2.1 PBPK model research procedures

The research included three parts:

1. PBPK model development and verification

2. PBPK model verification

3. PBPK model simulation

3.2.2 Softwares and hardwares

Simcyp® (Version 18, Certara, Sheffield, UK) was used in model analysis. Excel (Version 2010, Microsoft, USA) was used in data analysis. GraphPad Prism (Version 8, GraphPad Software, San Diego, CA, USA) was used in graph plotting

3.2.3 PBPK model development

The developed hydroxychloroquine PBPK model (version 1.0) were published in our previous study [1].

3.2.4 PBPK model verification

The Simcyp Simulator trial design was set to match population demographics (including ethnicity, age, sex), as well as drug administration and blood collection time points collected from the clinical studies. A population simulation was generated including 10 trials with 10 subjects in each trial. Predicted AUC and C_{max} were compared with clinical observations to assess the predictive performance of the PBPK model. Evaluation criteria: 1) the observed value is within the 90% confidence interval of the predicted value; 2) the ratio of AUC and C_{max} predicted value versus the observed value is within 2-fold, that is, $0.5 \leq ratio \leq 2.0$.

The validation results were published in our previous study [1].

3.2.7 Application of PBPK model

The default population library of “Sim-Healthy Volunteers”, “Sim-Chinese Healthy Volunteer”, “Sim-RenalGFR_30-60” in Simcyp® (Version 18, Certara, Sheffield, UK) were used in simulation. A population simulation was generated

including 10 trials with 10 subjects in each trial.

The simulation scenarios for hydroxychloroquine is shown in Table 1. Caucasians healthy, Caucasians renal injury with GFR 30-60 mL/min, and Chinese healthy population were simulated respectively.

Table 1: simulation scenarios for hydroxychloroquine

<table>
<tr><th>No.</th><th>Dose regimen</th><th>Duration</th></tr>
<tr><td>1</td><td>155 mg base daily for 14 days, (total dosing 14 times)</td><td rowspan="3">21 days</td></tr>
<tr><td>2</td><td>300 mg base on day 1, day 2, and day 3 then 155 mg base daily for 11 days (total dosing 14 times)</td></tr>
<tr><td>3</td><td>300 mg base daily for 14 days (total dosing 14 times)</td></tr>
</table>

4. Results and conclusions

4.1 PBPK model development and verification

The developed hydroxychloroquine PBPK model (version 1.0) were published in our previous study [1].

4.2 Simulation results

Mean PK profiles of HCQ in plasma, blood and lung of the three conditions in Chinese healthy population are shown in Figures 1, 2, 3.

Mean PK profiles of HCQ in plasma, blood and lung of the three conditions in Caucasian healthy population are shown in Figures 4, 5, 6.

Mean PK profiles of HCQ in plasma, blood and lung of the three conditions in Caucasian renal injury patients with GFR 30-60 mL/min are shown in Figures 7, 8, 9.

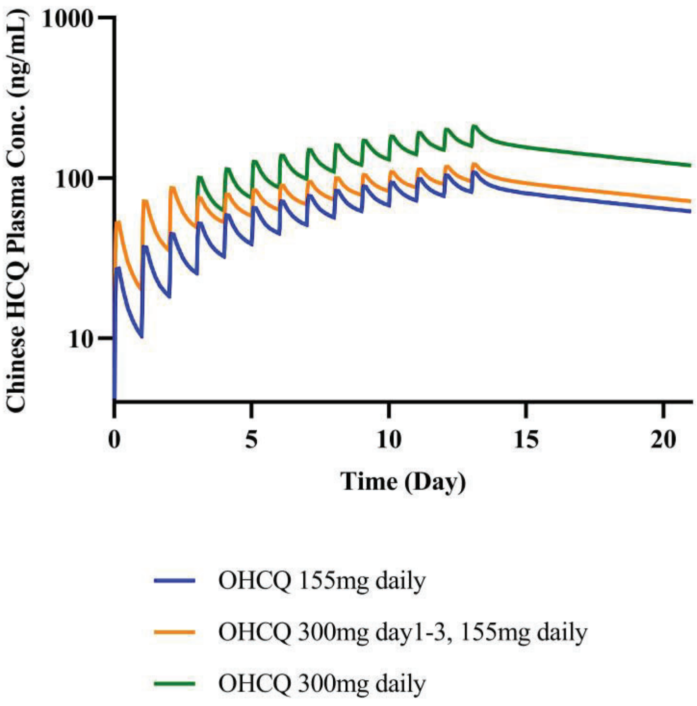

Figure 1: Simulated mean PK profile of HCQ in plasma in Chinese healthy populations.

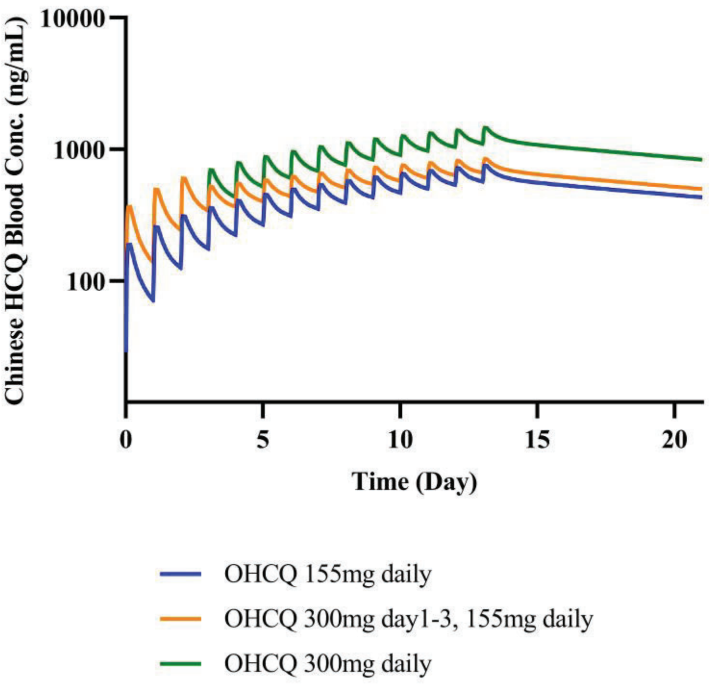

Figure 2: Simulated mean PK profile of HCQ in blood in Chinese healthy populations.

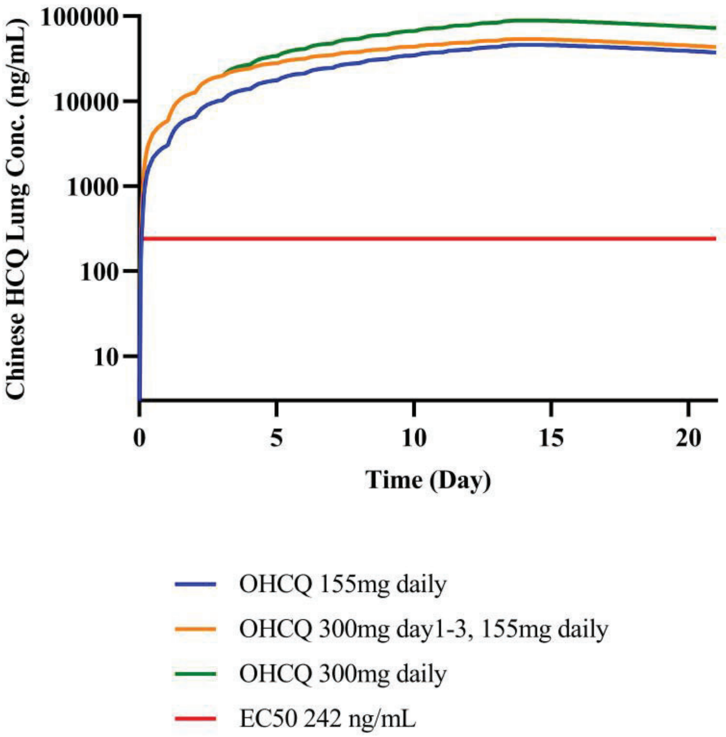

Figure 3: Simulated mean PK profile of HCQ in lung in Chinese healthy populations.

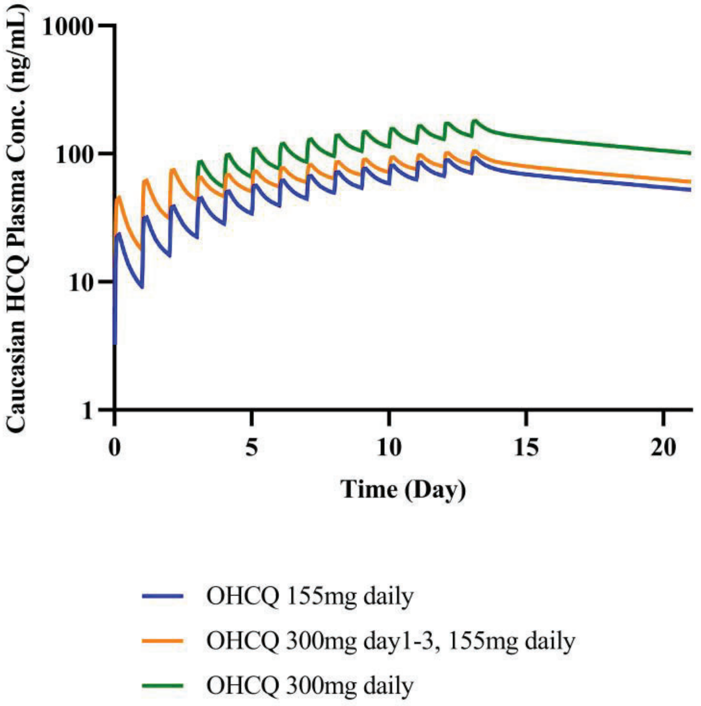

Figure 4: Simulated mean PK profile of HCQ in plasma in Caucasian healthy populations.

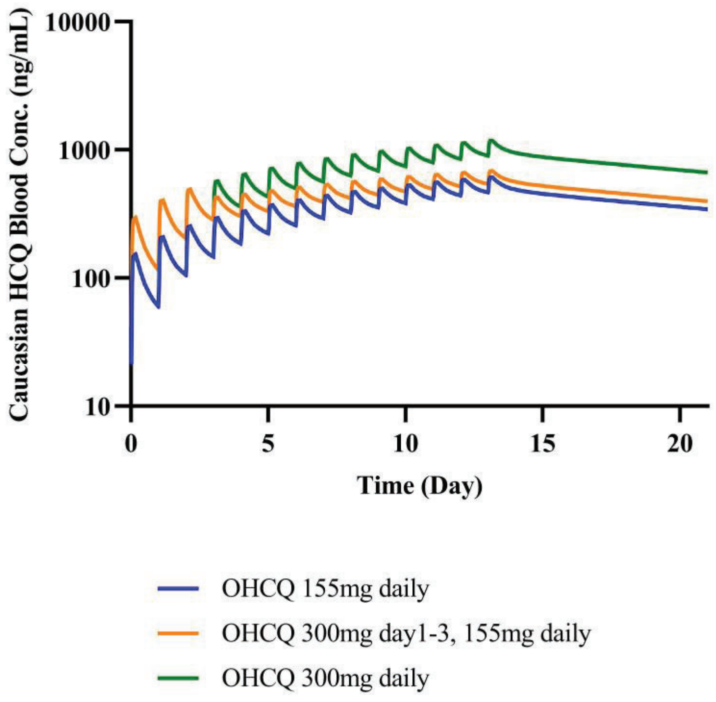

Figure 5: Simulated mean PK profile of HCQ in blood in Caucasian healthy populations.

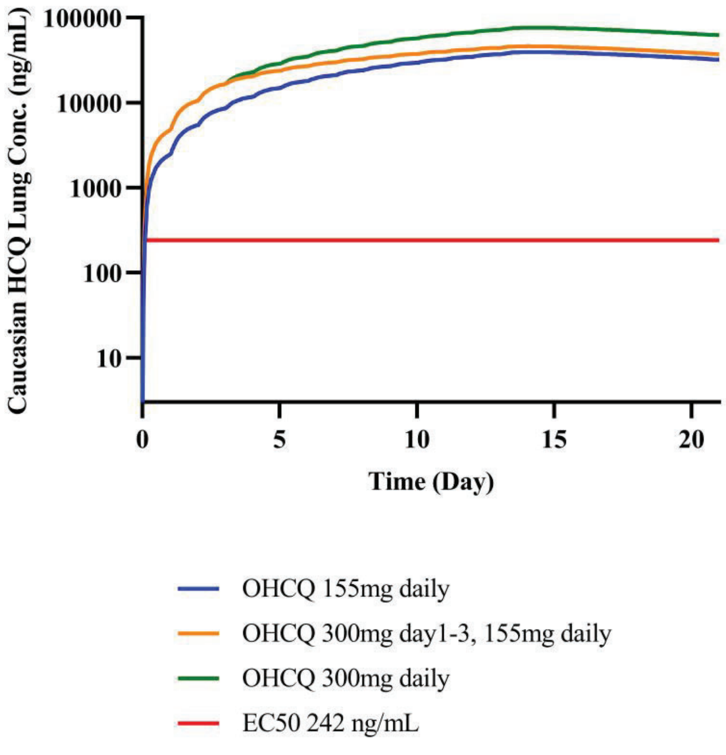

Figure 6: Simulated mean PK profile of HCQ in lung in Caucasian healthy populations.

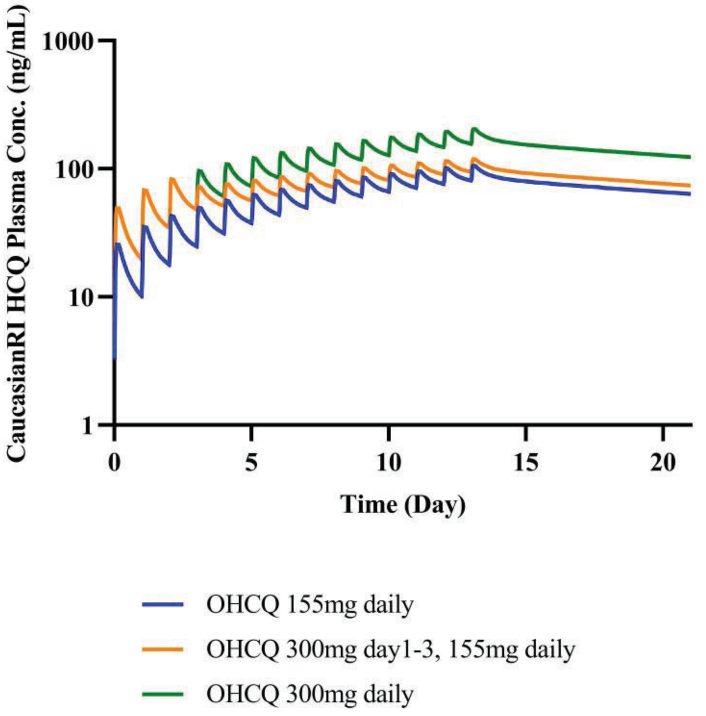

Figure 7: Simulated mean PK profile of HCQ in plasma in Caucasian renal injury patients with GFR 30-60 mL/min.

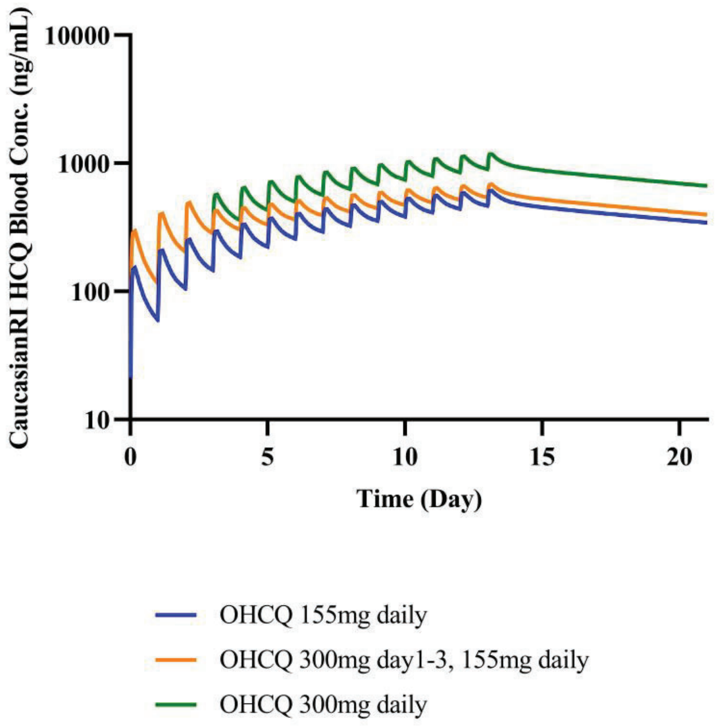

Figure 8: Simulated mean PK profile of HCQ in blood in Caucasian renal injury patients with GFR 30-60 mL/min.

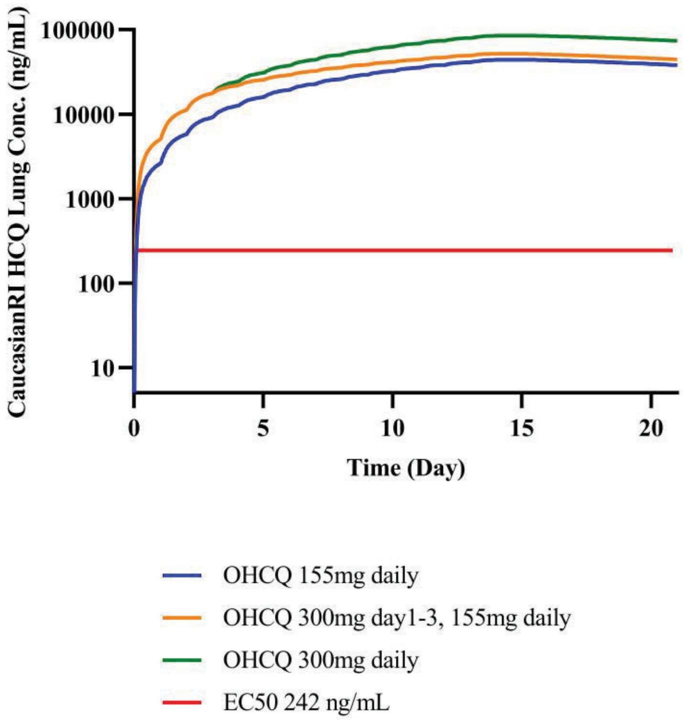

Figure 9: Simulated mean PK profile of HCQ in lung in Caucasian renal injury patients with GFR 30-60 mL/min.

5. Appendix

Individual simulations:
The following xls files are available up on request.
HCQ_LOW_Chinese_155 mg QD for 14 days.xlsx
HCQ_LOW_Caucasian_155 mg QD for 14 days.xlsx
HCQ_LOW_CaucasianRI (GFR 30-60) _155 mg QD for 14 days.xlsx
HCQ_MIDS_Chinese_300 mg QD D1-3 155mg for 11 days.xlsx
HCQ_MIDS_Caucasian_300 mg QD D1-3 155mg for 11 days.xlsx
HCQ_MIDS_CaucasianRI (GFR 30-60)_300 mg QD D1-3 155mg for 11 days.xlsx
HCQ_HIGH_Chinese_300 mg QD for 14 days.xlsx
HCQ_HIGH_Caucasian_300 mg QD for 14 days.xlsx
HCQ_HIGH_CaucasianRI (GFR 30-60)_300 mg QD for 14 days.xlsx

References

[1] Xueting Yao FY, Miao Zhang CC, Baoyin Huang PN, et al. In Vitro Antiviral Activity and Projection of Optimized Dosing Design of Hydroxychloroquine for the Treatment of Severe Acute Respiratory Syndrome 2 Coronavirus 2 (SARS-CoV-2). Clin Infect Dis. 2020. ciaa237.

Appendix 6: Pharmacokinetic Sample Collection and Analysis

Collection of Blood

Skin puncture

1. Before skin puncture, the participant should warm his/her hands. The finger is massaged anterogradely to enrich the blood flow toward the puncture site.
2. Clean the skin of the palmar side of the tip of the distal phalanx of the third or fourth finger of the non-writing hand with a suitable disinfectant, for example, 70% isopropyl alcohol. Puncture the skin by a single-use safety lancet. The finger should be held in such a position that the gravity facilitates the collection of blood from the fingertip.
3. When collection of capillary blood by skin puncture is complete, place a bandage on the fingertip.

Preparation of Blood Spots

Preparation from blood collected by skin puncture

1. Wipe off the first drop of blood with a gauze pad because it may contain excess tissue fluids. Massage the finger again to increase blood flow at the puncture site. Transfer the following drop to one of the circles of a filter card without touching the surface directly with the fingertip. Allow the blood to be soaked into the texture of the filter by capillary forces only.
2. Let the next large drop of capillary blood form on the fingertip and collect it in the next circle. Continue this procedure until all necessary circles are filled or blood flow stops.
3. Do not squeeze or “milk” the finger excessively if the blood flow is not sufficient to fill all the required circles of the filter card. If blood flow stops, place a bandage on the fingertip. Perform a second skin puncture on another finger if more blood is needed for the examination.
4. For blood obtained by venipuncture, use syringe to apply approximately 100 µL of blood on the filter paper.

Drying of Blood Spots

To dry the blood spots, put the filter cards on a clean paper towel and let them dry, preferably overnight (but for at least 4 hours), at room temperature in the absence of any external source of heat. When the drying process is complete, the blood spots have a uniformly dark brownish color and no red areas are visible anymore.

Storage and Transportation of Dried Blood Spots (DBS)

NOTE: Processing of the blood spots can be interrupted after drying. The filter cards can now be stored.

1. For storage, put the filter paper card in a single, gas-impermeable zipper bag, containing 1 to 2 desiccant sachets to protect the specimens from moisture. Optionally, add a humidity indicator card.
2. Store at ambient temperature until the DBS card is collected.

On the filter paper, the participant should record the following:

1. Date and time of sample
2. Date and time of last dose

Samples that appear to be collected according to the schedule of activities, which have the required amount of blood for a 5- to 6-mm punch and have the minimal required information (1 through 2 above), will be processed for hydroxychloroquine (and metabolite) concentration.
